# Overlapping motifs on the herpes viral proteins ICP27 and ORF57 mediate interactions with the mRNA export adaptors ALYREF and UIF

**DOI:** 10.1038/s41598-018-33379-x

**Published:** 2018-10-09

**Authors:** Richard B. Tunnicliffe, Xiaochen Tian, Joanna Storer, Rozanne M. Sandri-Goldin, Alexander P. Golovanov

**Affiliations:** 1 Manchester Institute of Biotechnology, School of Chemistry, Faculty of Science and Engineering, The University of Manchester, Manchester, M1 7DN UK; 2 Department of Microbiology and Molecular Genetics, School of Medicine, University of California, Irvine, CA 92697-4025 USA

## Abstract

The TREX complex mediates the passage of bulk cellular mRNA export to the nuclear export factor TAP/NXF1 via the export adaptors ALYREF or UIF, which appear to act in a redundant manner. TREX complex recruitment to nascent RNA is coupled with 5′ capping, splicing and polyadenylation. Therefore to facilitate expression from their intronless genes, herpes viruses have evolved a mechanism to circumvent these cellular controls. Central to this process is a protein from the conserved ICP27 family, which binds viral transcripts and cellular TREX complex components including ALYREF. Here we have identified a novel interaction between HSV-1 ICP27 and an N-terminal domain of UIF *in vivo*, and used NMR spectroscopy to locate the UIF binding site within an intrinsically disordered region of ICP27. We also characterized the interaction sites of the ICP27 homolog ORF57 from KSHV with UIF and ALYREF using NMR, revealing previously unidentified binding motifs. In both ORF57 and ICP27 the interaction sites for ALYREF and UIF partially overlap, suggestive of mutually exclusive binding. The data provide a map of the binding sites responsible for promoting herpes virus mRNA export, enabling future studies to accurately probe these interactions and reveal the functional consequences for UIF and ALYREF redundancy.

## Introduction

The production of mature messenger RNA (mRNA) in metazoans involves a dynamic series of protein assemblies that orchestrate transcription through to translation. Within the cell nucleus, a pre-initiation complex (PIC) is assembled on promoter regions; the PIC is composed of transcription factors, DNA helicase and RNA polymerase II (RNAPII)^1^. Immediately following initiation of transcription by RNAPII the emerging 5’ end of the nascent transcript is stabilized by addition of a 7-methylguanosine cap, later important in translation^2,3^. Then as elongation of the RNA proceeds, splicing factors are recruited mediating the co-transcriptional removal of introns; this process occurs in proximity to sub-nuclear speckles^4^. Finally, polyadenylation of the 3’-termini of the transcript occurs yielding mature mRNA^5^. The subsequent nuclear export of the mRNA to the cytoplasm via the transcription-export (TREX) complex and the nucleoporin-interacting export receptor (TAP/NXF1) is coupled with splicing and the release from nuclear speckles^4,6–8^. The conventional TREX model contains a hexameric protein assembly termed the THO complex^7^, plus UAP56 an RNA helicase and ALYREF (also called Aly or REF)^9–11^. Significantly, UAP56 catalyzes the initiation of splicing^12,13^ and also recruits ALYREF to the RNA^14,15^, which in turn interacts with the nuclear export protein NXF1/NXT1 (also called TAP/p15) simultaneously transferring the mRNA to the latter^16^. More recently, in addition to ALYREF, other mRNA export adaptors that are recruited by UAP56 have been identified such as UIF, which provides redundancy in this step of TREX assembly^17^. Other proteins have also been proposed to be components of the TREX complex including the UAP56 binding partners CHTOP and CIP29^18,19^, plus POLDIP3^20^. TAP/NXF1 interacts with the nuclear pore and mediates passage of spliced mature mRNAs into the cytoplasm for subsequent translation^6^. Thus ALYREF and the functional analogue UIF perform a central role in the TREX complex, which couples transcription and splicing through to nuclear mRNA export. The structures of human ALYREF and the murine homolog REF2-I, plus their interaction sites for UAP56, TAP/NXF1 and RNA have been determined^21,22^. In contrast, similar structural information and binding site mapping is lacking for UIF, as only the short UAP56 binding motif (UBM) has been identified by homology with ALYREF^17^.

**Figure 1:**
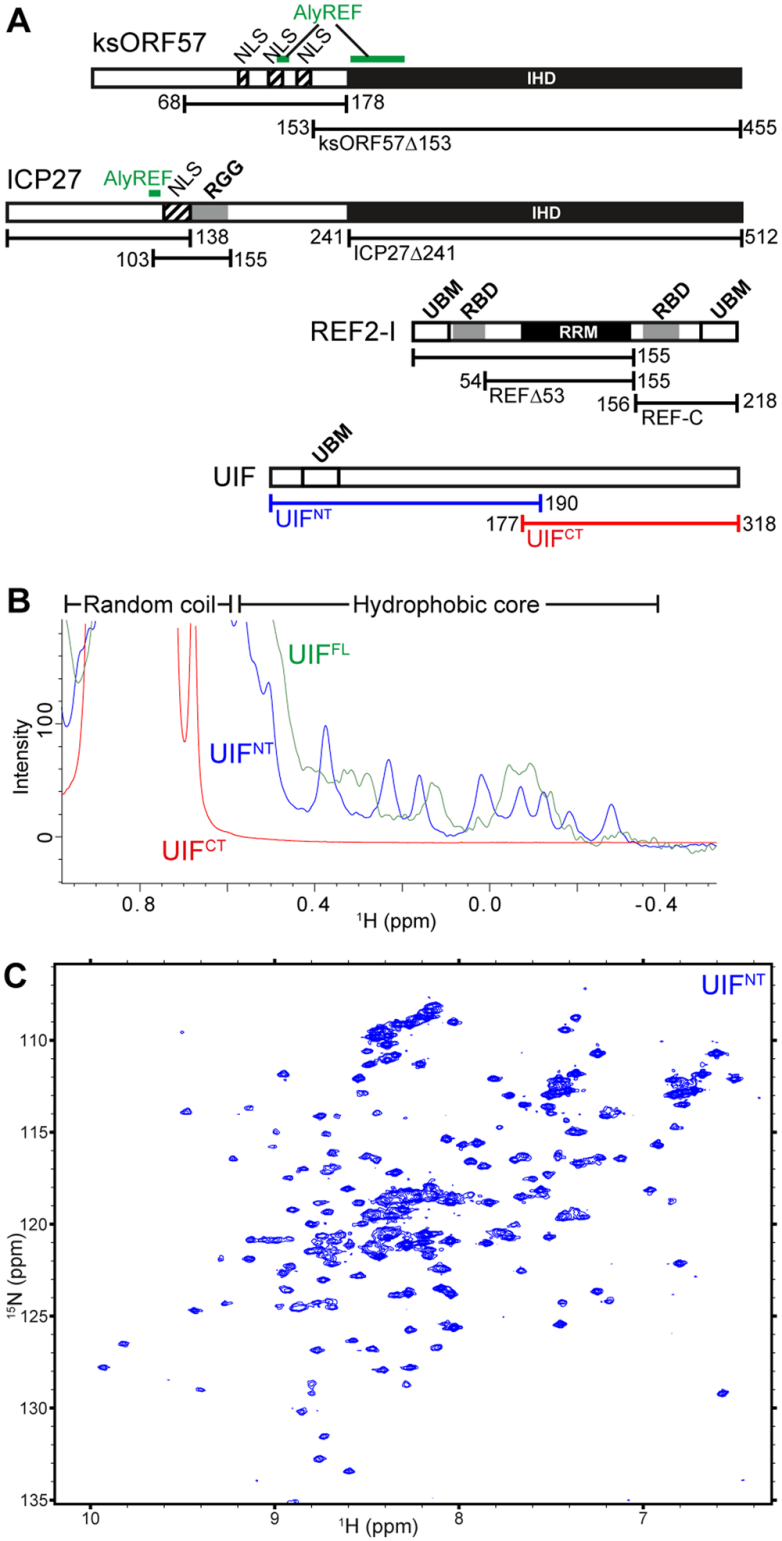
Summary of protein constructs and characterization of the protein folding of UIF by NMR. (**A**) Schematic of proteins, their domains and functional motifs employed in this study. Known folded domains are indicated as black filled boxes (labeled IHD for ICP27-homology domain, and RRM for RNA-recognition motif). ALYREF binding sites are labeled green and RNA binding motifs grey boxes, nuclear localization sequences (NLS) are shown as hatched boxes, also UAP56-binding motifs (UBM) are labeled. (**B**) 1D ^1^H spectra of UIF constructs reveal upfield methyl signals characteristic of a folded globular protein in UIF^FL^ and UIF^NT^ but not UIF^CT^. (**C**) ^15^N HSQC spectrum of UIF^NT^ contains well-dispersed backbone amide signals indicative of a globular folded protein.

Considering the close connection between splicing and mRNA export, it is perhaps somewhat surprising that some viruses such as herpes viruses do not have introns within the majority of their genes. Thus, herpes viruses must circumvent cellular controls to trick the host cell into producing viral proteins and therefore facilitate viral replication. Herpes viruses have evolved a highly effective mechanism for the maturation of non-spliced viral transcripts, central to which is a conserved multifunctional protein, the archetype being ICP27 from herpes simplex virus 1 (HSV-1)^23^. Members of the ICP27 family are found in all herpes viruses that have been sequenced, and the region of conservation is a globular ICP27-homology domain (IHD), which is responsible for dimerization^24–26^. The IHD is located at the C-terminus of ICP27, whereas the N-terminus forms an intrinsically disordered region (IDR). This structural architecture, a disordered N-terminus plus globular C-terminal homo-dimer, is present in ICP27 homologs in alpha and gamma herpes viruses, whereas in beta herpes viruses the central globular domain (that mediates tetramerization) is flanked by disordered regions on both N- and C-termini^25–31^. Functionally significant motifs within ICP27 have been identified in the N-terminal IDR, such as an RGG-box that is necessary for interactions with viral transcripts^32–34^. Additionally there is also a nuclear localization sequence (NLS) necessary for nuclear-cytoplasmic shuttling^34–36^ and also an adjacent binding motif for ALYREF; interaction with the latter enhances the efficiency of mRNA export^37–40^ (Fig. 1A). ICP27 can also interact with cellular proteins such as RNA polymerase II via its C-terminal domain^41^, plus TAP/NXF1^37,38,42^ and cellular splicing proteins^43,44^. Therefore ICP27 is promiscuous in its ability to interact with numerous cellular binding partners that have roles throughout the process of mRNA maturation and nuclear export.

The ICP27 homolog ORF57 from herpes virus saimiri (HVS) is a close relative of the Kaposi sarcoma herpes virus (KSHV) ORF57 protein (ksORF57)^45–47^. Like ICP27, HVS ORF57 (hvsORF57) also contains an ALYREF binding motif located within an N-terminal IDR, and the solution structure of the binding interface has been determined^40,48,49^. The interaction sites on hvsORF57 for RNA and ALYREF partially overlap, as mapped directly by NMR spectroscopy, thus the interaction of ALYREF with an ORF57-RNA complex facilitates the transfer of RNA to the cellular protein^48^. Within ksORF57 the ALYREF binding site was probed thus far only by deletion mutagenesis and a region of the globular domain, later defined as a PxxP motif (ksORF57 residues 208–211) has been implicated^50,51^. However mutagenesis studies targeting the ksORF57 NLS located within the N-terminal IDR also resulted in weakening of the interaction with ALYREF to background levels without apparently affecting the cellular localization of ksORF57^52^, which could indicate that this region may contribute to the interaction with ALYREF. Recently other cellular mRNA export adaptors have been identified as binding partners for ksORF57 in addition to ALYREF, specifically UIF, CHTOP, CIP29, RBM15 and OTT3^53–55^. The ability to interact with these cellular proteins provides redundancy and likely enhanced efficiency for viral mRNA accumulation and export. Such redundancy has not been described for other herpes virus ICP27 homologs to date.

In order to explore if mRNA export adaptor interaction redundancy is also a feature within HSV-1, here we investigated if ICP27 is able to interact with UIF, with our data revealing that these proteins do interact *in vivo*. We also determined an interaction site *in vitro* by solution NMR, a region that partially overlaps with the ALYREF binding site in the N-terminal IDR of ICP27. Previous studies of interactions between the N-terminal IDRs of HSV-1 ICP27 and hvsORF57 identified common binding characteristics for ALYREF binding, despite low sequence homology. Therefore, here we used solution NMR to investigate if similar interactions occur between the ksORF57 protein N-terminal IDR, and the cellular proteins ALYREF and UIF, revealing binding sites that in common with ICP27, also partially overlap. These data thus identified new interaction sites for UIF with ICP27 and ksORF57, and also an additional ALYREF binding site in the latter. Together our findings indicate that distantly related ICP27 homologs are similarly able to utilize the redundancy present in cellular mRNA export adaptors.

## Results

### Subdivision of UIF into fragments

No structural information is currently available for UIF, and it lacks significant sequence homology with protein domains of known structure. Therefore at the start we used *in silico* predictions of secondary structure, solvent accessibility and disordered regions based on the primary sequence using the PredictProtein program^56^. The data was indicative of a primarily intrinsically disordered N-terminus and a structured C-terminus (Fig. S1). Based on these predictions three constructs were cloned and expressed comprising full length and the N- and C-terminal regions of UIF, specifically residues 1–318, 1–190 and 177–318 (here named UIF^FL^, UIF^NT^ and UIF^CT^ respectively) (Fig. 1A).

### UIF contains a folded domain

In order to investigate experimentally the presence of a structured globular domain within the UIF constructs chosen, we expressed the proteins in *E*. *coli*, then purified and analyzed their NMR spectra. The UIF^FL^ and UIF^NT^ proteins were expressed and purified from the soluble fraction, whereas UIF^CT^ was expressed in inclusion bodies and therefore purified in denaturing conditions and re-folded (re-solubilized) into a native buffer. 1D ^1^H NMR spectra were used to assess protein folding. UIF^FL^ and UIF^NT^ spectra contained features indicative of a presence of a folded globular domain, such as upfield shifted methyl groups characteristic of a hydrophobic core and dispersion of backbone amides, however UIF^CT^ lacked such features and therefore appeared to not be folded even after the refolding procedure (Figs 1B and S2). HSQC spectra of ^15^N labeled UIF^NT^ also further corroborated this analysis clearly showing dispersed backbone amide signals characteristic of a globular protein (Fig. 1C). It is plausible that the C-terminal domain may fold correctly only *in vivo* and in our experiments was either destabilized by truncation, lacked a crucial co-factor, post-translational modifications or chaperones, or could not be refolded properly *in vitro* from the denatured state needed for protein purification. The NMR analysis of UIF therefore indicated that overall UIF does contain at least one globular domain and, contrary to sequence-based predictions, the folded region is contained within residues 1–190, either independently, or as a part of a larger 3D structure.

### HSV-1 ICP27 interacts with an N-terminal fragment of the cellular RNA export adaptor protein UIF

**Figure 2:**
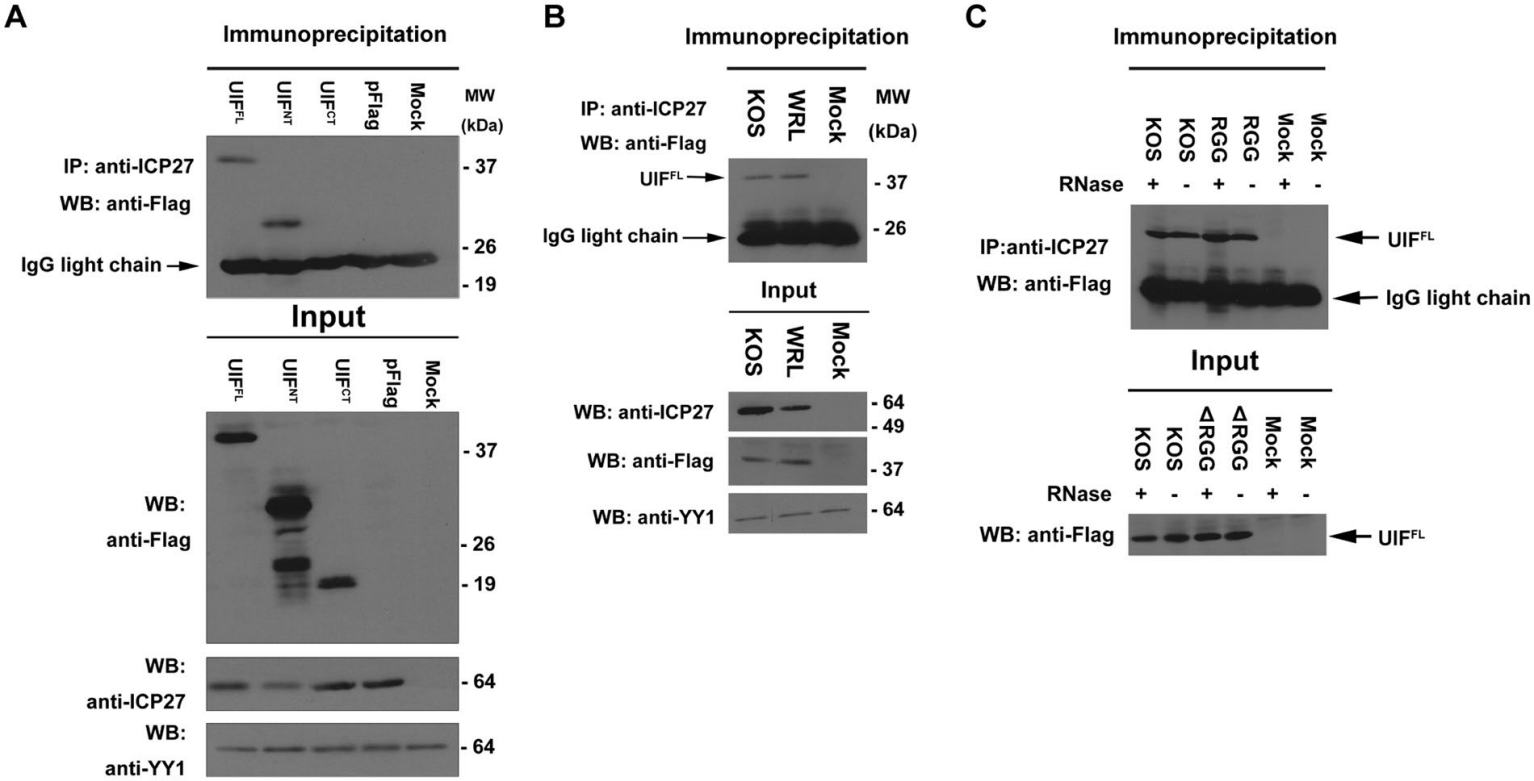
ICP27 interacts with UIF in co-immunoprecipitation assays. (**A**) HeLa cells were transfected with Flag-tagged UIF^FL^, UIF^NT^, UIF^CT^ or Flag-tagged pUC18 plasmid DNA as indicated. Cells were infected 24 h after transfection with WT HSV-1 or were mock infected and 8 h later cell lysates were immunoprecipitated with anti-ICP27 antibody. Western blots were probed with anti-Flag antibody. Samples of each lysate were analyzed in parallel with the immunoprecipitated samples, and the Western blot is labeled input. The blot was probed with anti-YY1 antibody as a loading control. (**B**) Cells were transfected with Flag-tagged UIF^FL^ plasmid DNA and were either mock infected or infected with WT HSV-1 KOS or WRL, in which the ALYREF binding site is mutated, as indicated, and immunoprecipitation was performed with anti-ICP27 antibody. Western blots were probed with anti-Flag, anti-ICP27 and anti-YY1 antibody as indicated. (**C**) Cells transfected with UIF^FL^ plasmid DNA were mock infected or infected with HSV-1 KOS or ΔRGG, in which the RGG box RNA binding domain is deleted. Cells lysates were either treated or were not treated with RNase as indicated. Immunoprecipitation was performed with anti-ICP27 antibody and western blots were probed with anti-Flag antibody.

To determine if ICP27 interacts with UIF during viral infection, Flag-epitope tagged UIF^FL^, UIF^NT^ and UIF^CT^ plasmid DNA were transfected into cells and 24 h later, cells were infected with HSV-1 KOS for 8 h. Cell lysates were immunoprecipitated with anti-ICP27 antibody and western blot analysis was performed with anti-Flag antibody (Fig. 2A). Flag-tagged constructs UIF^FL^ and UIF^NT^ were co-immunoprecipitated with ICP27, however Flag-tagged UIF^CT^ did not interact with ICP27 (Fig. 2A). Next, we determined if the ALYREF binding site triple mutant W105A, R107A and L108A (the ‘WRL’ mutant)^39,40^ was able to interact with UIF. Cells transfected with UIF^FL^ were infected with WT KOS or WRL. UIF was efficiently co-immunoprecipitated with the WRL mutant ICP27 indicating that binding sites of ALYREF and UIF are not identical (Fig. 2B).

Because ICP27 and UIF both bind to RNA, we next set out to determine if the interaction between UIF and ICP27 was mediated by RNA. HSV-1 KOS and ICP27 mutant ΔRGG, which has a deletion of the ICP27 RGG box required for RNA binding, were used to infect cells transfected with Flag-UIF. Immunoprecipitation was performed on lysates that were or were not treated with RNase (Fig. 2C). UIF was co-immunoprecipitated with WT ICP27 and ΔRGG in both the presence and absence of RNase indicating that the interaction between ICP27 and UIF was not mediated by RNA binding. Thus, ICP27 interacts with UIF during viral infection and the UIF interaction site is located within the N-terminal half of UIF.

### Mapping the interaction of UIF^NT^ with ICP27

**Figure 3:**
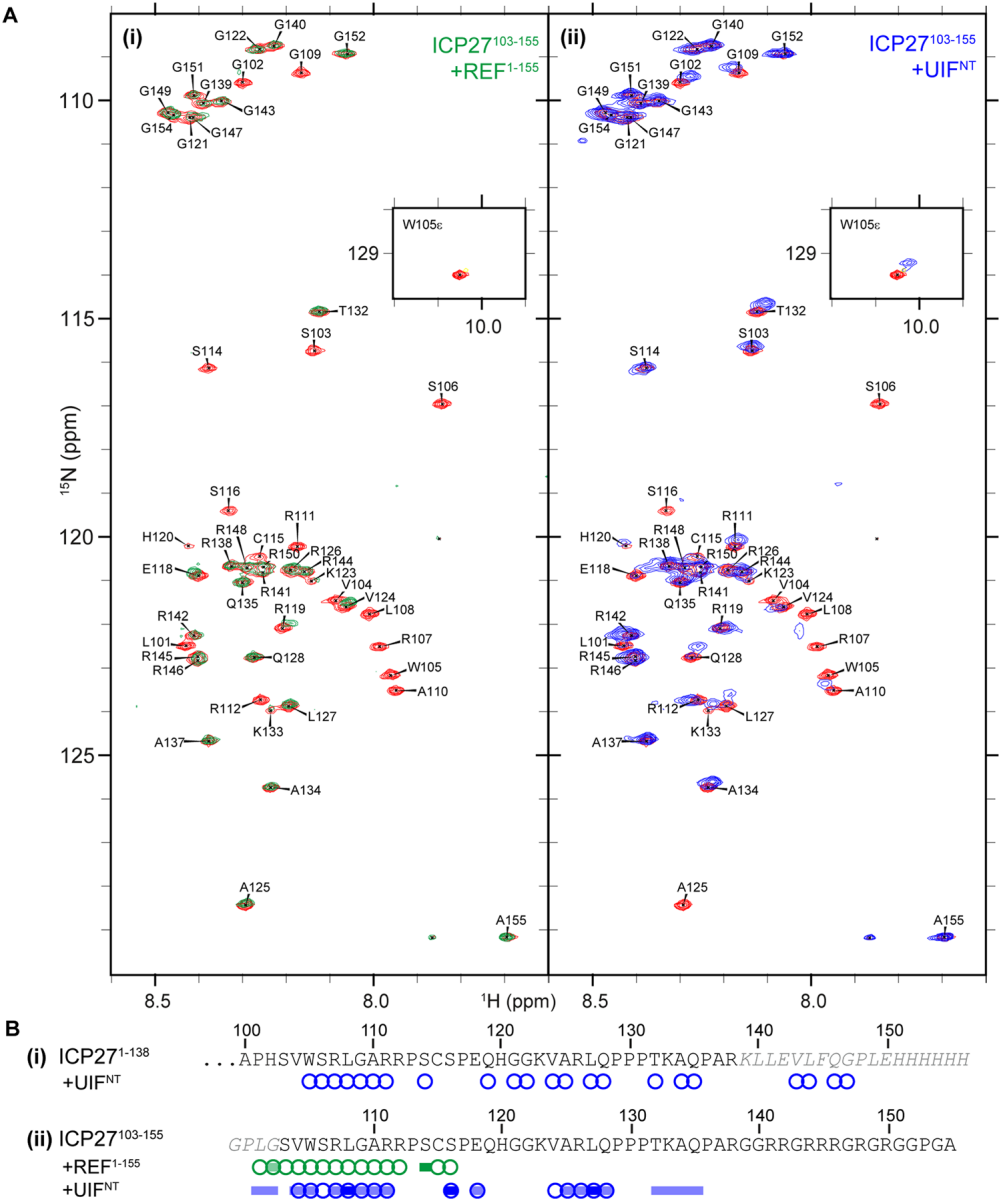
NMR mapping of the UIF^NT^ interaction with ICP27 intrinsically disordered N-terminus. (**A**) ^15^N HSQC of ICP27^103–155^ with signal assignments, spectra in the absence of binding partner is colored red overlaid with spectra with equimolar: (i) REFΔ53 colored green and (ii) UIF^NT^ colored blue. (**B**) Signal perturbations mapped onto the sequence of ICP27 constructs (i) ICP27^1–138^ and (ii) ICP27^103–155^, lettering in grey italics are non-native residues introduced during cloning. Residues with broadened signals are indicated by circles, with moderate and large shifts indicated by light and dark blocks, respectively, colored as in panel A.

We have previously used NMR spectroscopy to determine the binding site between the RRM-domain of ALYREF and the N-terminal intrinsically disordered region of ICP27^40^. These experiments used murine REF2-I protein constructs, a protein that has been used extensively in previous studies of ALYREF protein function^11,16,21,40,48,50,57^. To determine if ICP27 interacts with UIF via a similar site as ALYREF/REF2-I, we used solution NMR to determine if the UIF binding site was located within an N-terminal region of aa1–138 of ICP27 (ICP27^1–138^). First the backbone amide signals of uniformly [^13^C,^15^N] labeled ICP27^1–138^ (with an additional C-terminal His_6_-tag) were assigned using standard triple resonance experiments. Nearly complete (97%) sequence-specific backbone signal assignment was achieved within residues 1–138, and the data was deposited into the BMRB (accession code: 27483). Then HSQC spectra of a ^15^N labeled sample of ICP27^1–138^ were acquired in the presence and absence of a stoichiometric amount of unlabeled UIF^NT^ (Fig. 3A). The addition of UIF^NT^ induced signal broadening in the majority of residues in the region 105–135, with several signals also affected from non-wild type C-terminal residues introduced in cloning (Figs 3B and S3). Interestingly the region perturbed by UIF^NT^ significantly overlaps with the binding site for ALYREF, residues 103–112. As a further control to investigate the possibility that the residues introduced during cloning were inadvertently mediating the interaction with UIF^NT^, the experiment was repeated with a different ICP27^103–155^ construct, extended at C-terminus with native sequence and lacking these non-wild type residues. We expressed and purified [^13^C,^15^N]-labeled ICP27^103–155^ and used standard triple resonance NMR experiments to assign the backbone amide signals to sequence positions; the data was deposited into the BMRB (accession code: 27341). Addition of UIF^NT^ to ^15^N-labeled ICP27^103–155^ resulted in similar perturbations to that observed as in ICP27^1–138^ construct, with the most significant signals changes within two patches aa104–111 and aa124–128 (Fig. 3B). Control spectra of ICP27^103–155^ were also acquired in the presence of REF^1–155^ (Fig. 3A,B) showing perturbations within the previously identified binding site (residues 103–112). The data therefore indicated that ALYREF and UIF bind to adjacent and partially overlapping binding sites on ICP27, possibly in a redundant manner. However, as the mutations of WRL triad residues critical for interactions with ALYREF^39,40^ did not block interactions with UIF, the two interactions are not equivalent.

### Mapping the interactions of ksORF57 with ALYREF

**Figure 4:**
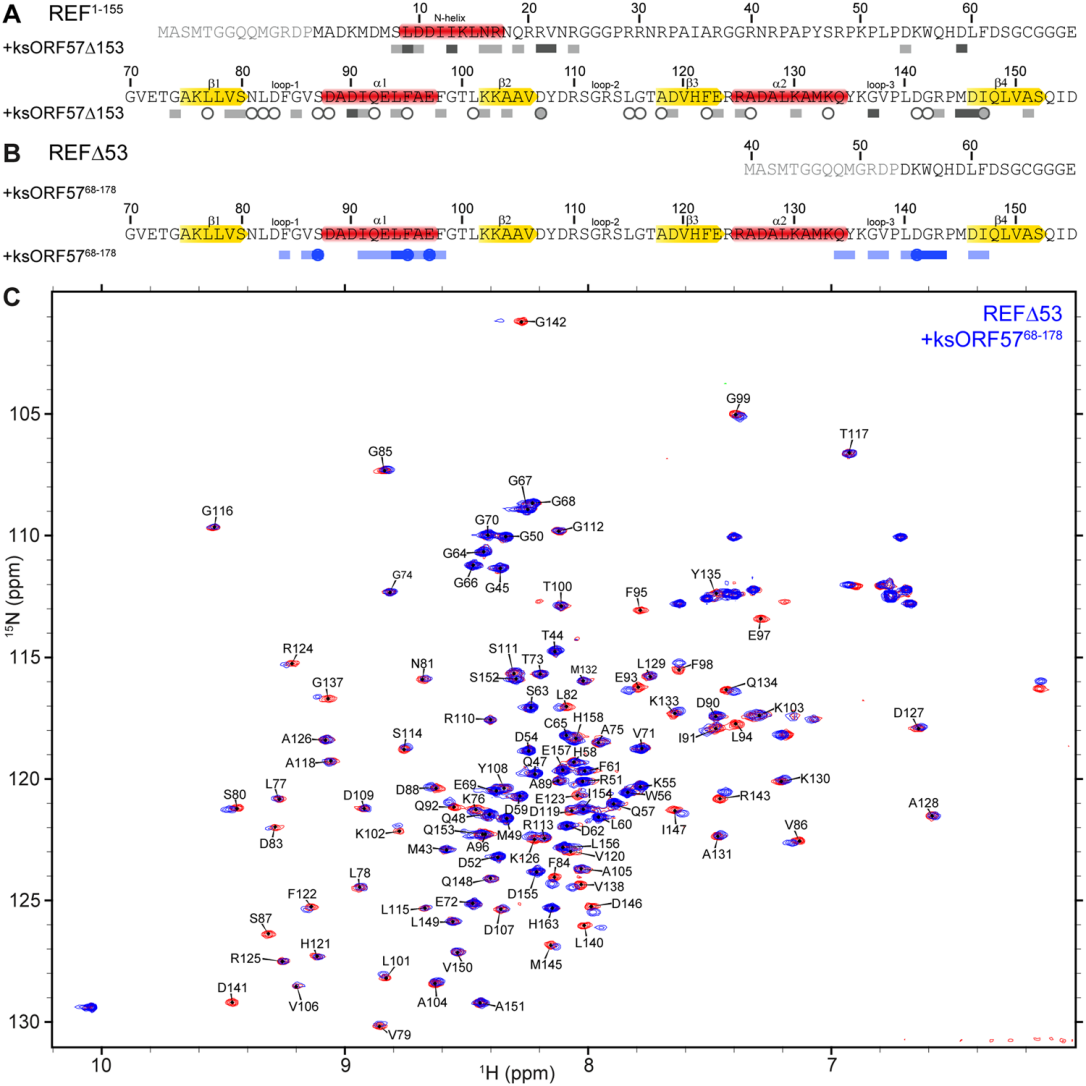
Mapping of ALYREF/REF2-I interaction with KSHV ORF57 by NMR. Perturbations in ^15^N HSQC spectra were used to map interactions of ^15^N-labeled REF2-I constructs with unlabeled ksORF57 constructs. (**A)** Signal shifts and intensity perturbations induced by ksORF57Δ53 on REF^1–155^ mapped onto its sequence. (**B**) Signal shifts and intensity perturbations induced by ksORF57^68–178^ on REFΔ53 mapped onto its sequence. Secondary structure elements are highlighted on the primary sequence; α-helix in red, β-sheet in yellow. Broadened residues indicated by circles and moderate and large shifts indicated by light and dark blocks, respectively. (**C**) Example spectrum of free ^15^N labeled REFFΔ53 (red) is overlaid with spectrum upon addition of ksORF57^68–178^ (blue) shows spectral perturbations assigned to RRM region of REF2-I.

An ALYREF interaction was previously detected with ksORF57, and deletion mutagenesis of ksORF57 indicated that residues 181–215 contained the ALYREF binding site^50,51^. Primary sequence alignment suggests that this binding site is within the globular ICP27-homology domain that is likely contained within the C-terminal residues 178–455 of ksORF57. Previous partial proteolysis experiments indicated a ksORF57 construct comprising residues 153–455 formed a stable protein fragment^27^, therefore we cloned, expressed and purified this fragment of ORF57, and named it ksORF57Δ153. Using NMR, we monitored signal shift and intensity perturbations within HSQC spectra of ^15^N-labeled REF^1–155^ and ^15^N REF-C (aa156–218) upon addition of unlabeled ksORF57Δ153, which resulted in substantial signal broadening within the RRM of REF^1–155^ which is suggestive of a direct interaction, whereas no significant changes were observed in the REF-C spectra (Figs 4A and S4–S6). This result corroborates the previously identified ALYREF binding region 181–215 including the PxxP motif (residues 208–211), which is present in the ksORF57Δ153 construct^50,51^. A control experiment where ICP27Δ241, comprising the folded domain of ICP27, was added to ^15^N-labeled REF^1–155^ was not indicative of any interaction (Figs S5C, S7). The data therefore suggests that the C-terminal domain of ksORF57 interacts with the RRM domain of ALYREF, while the corresponding folded domain of ICP27 does not interact, despite sequence homology between these viral proteins.

Previously an ALYREF binding site on herpesvirus saimiri (HVS) ORF57, a close homolog of KSHV ORF57, was located within residues 103–120, part of an intrinsically disordered region (IDR)^40,48^. In order to determine if there is an analogous interaction site for ALYREF in the N-terminal IDR of KSHV ORF57, we expressed and purified KSHV ORF57 residues 68–178 (ksORF57^68–178^). Addition of equimolar ksORF57^68–178^ to ^15^N-labeled REF^1–155^ induced perturbations within the RRM domain (Fig. S8). To explore this interaction further, the experiment was repeated with a shorter construct with improved spectral quality, namely ^15^N-labeled REFΔ53, to which unlabeled ksORF57^68–178^ was added to a 5-fold molar excess (Fig. 4B,C). The chemical shift changes were mapped to the RRM helices α1 + α2 and loops 1 + 2 (Fig. 4B). This interaction site on the RRM of ALYREF resembled that previously observed for binding of the N-terminal IDR of HVS ORF57 and also HSV-1 ICP27^40,48^.

**Figure 5:**
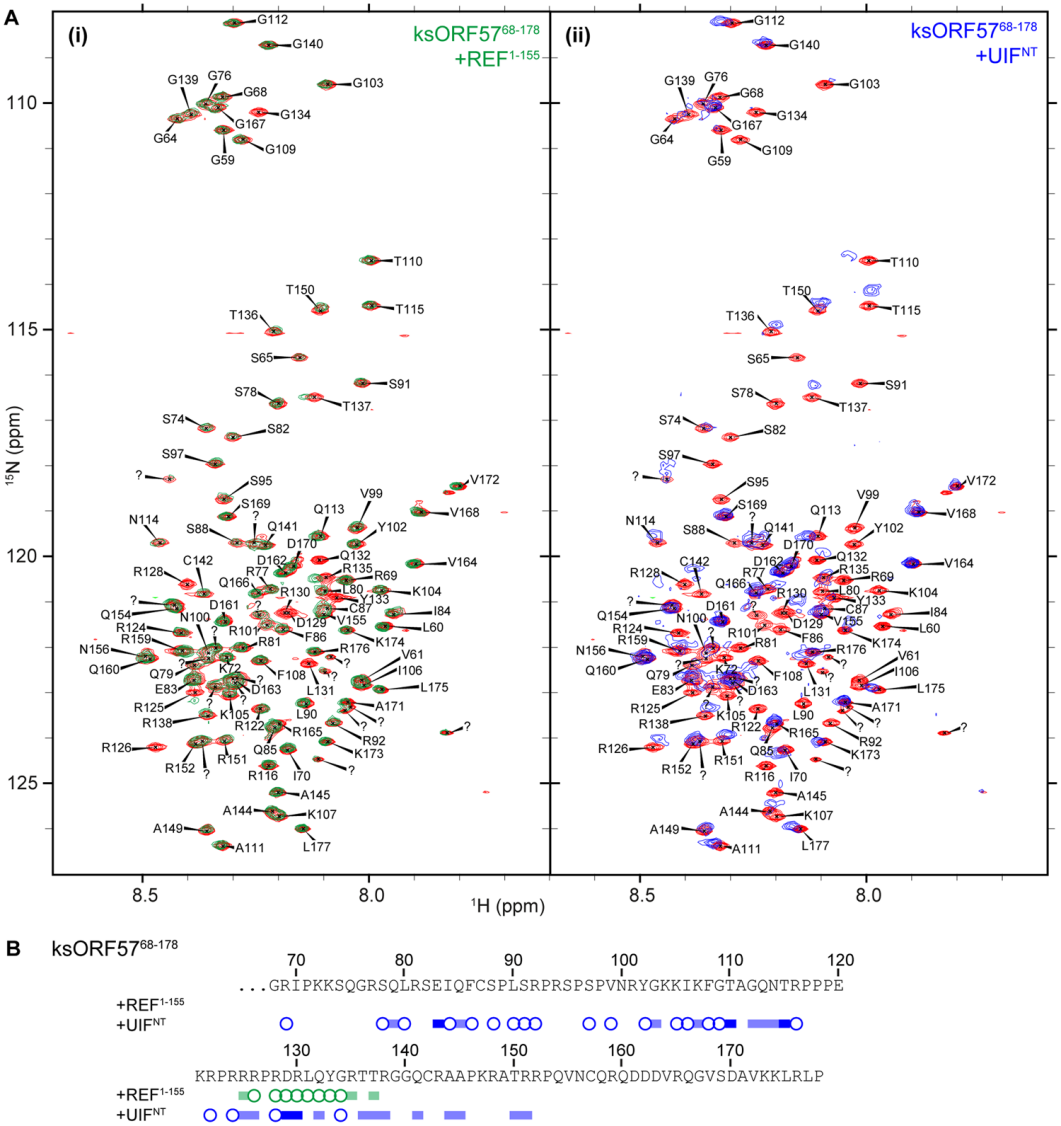
KSHV ORF57 residues 68–178 interaction with UIF and ALYREF mapped by NMR. (**A**) Perturbations in ^15^N HSQC spectra were used to map interactions of ^15^N-labeled ksORF57^68–178^ with unlabeled constructs: (i) ksORF57^68–178^ with REF^1–155^ (free in red, bound in green), (ii) ksORF57^68–178^ with UIF^NT^ (free in red, bound in blue). (**B**) Signal shifts and intensity perturbations induced by REF and UIF mapped onto the sequence of ksORF57 from analysis of spectra in panel A. Broadened residues indicated by circles and moderate and large shifts indicated by blocks.

**Figure 6:**
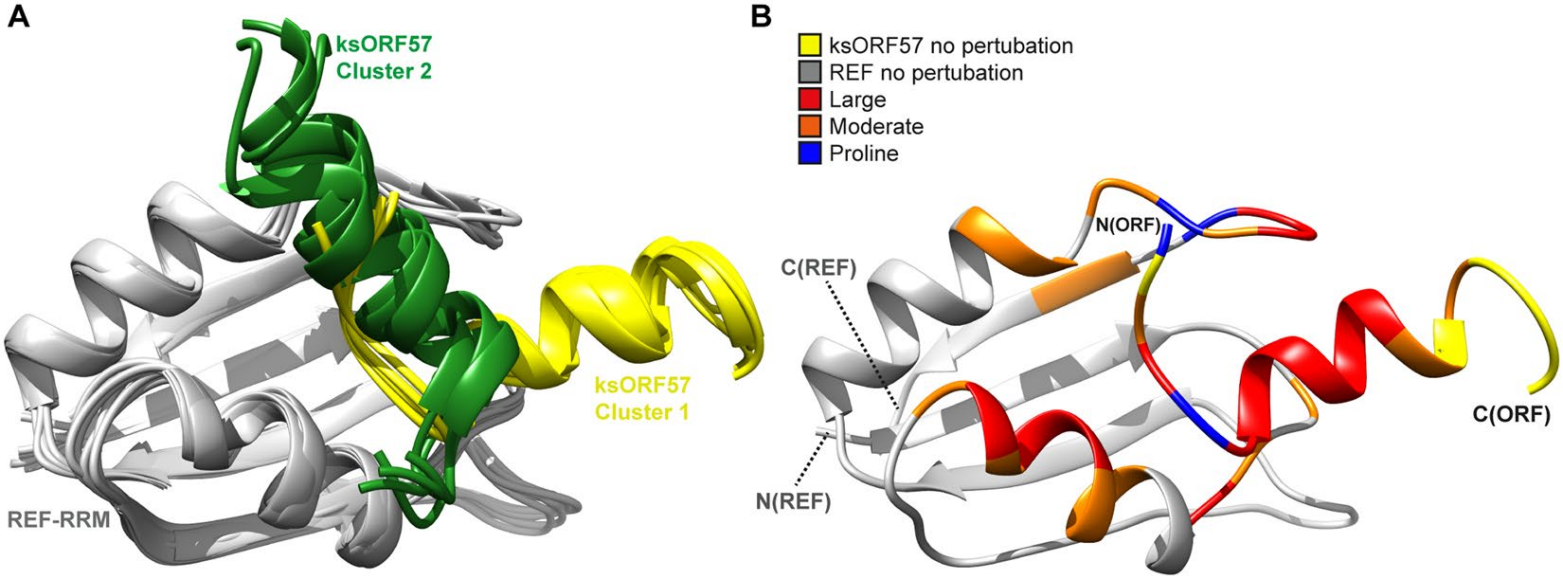
Haddock structural model of ALYREF RRM domain (from REF2-I) interaction with N-terminal domain of ksORF57 and comparison with HVS ORF57-REF structure. (**A**) Haddock structural model of the REF-ksORF57 complex provided two major conformational clusters for ksORF57 residues 123–140. (**B**) Backbone amide signal perturbations mapped onto a representative structure of REF-ksORF57.

To determine the binding site location for ALYREF within ksORF57^68–178^, we expressed and purified ^15^N labeled ksORF57^68–178^, and it was possible to assign 80% of the backbone amides using TOCSY and NOESY-HSQC spectra (the assignment data was deposited into the BMRB, accession code 27484). Addition of unlabeled REF^1–155^ induced signal broadening most significantly for residues 126–134 of ksORF57, thus identifying a short binding motif (Fig. 5Ai,B). The data therefore suggests that a common binding mode for the ALYREF RRM is shared by the N-terminal IDR domains of both HVS and KSHV ORF57 proteins, as well as by HSV-1 ICP27. To illustrate the mode of interaction, a structural model of this ksORF57-ALYREF complex was assembled using Haddock and the chemical shift perturbation mapping data (Fig. 6)^58^. The region of ksORF57 that contacted REF was predicted to be α-helical and two orientations of this helix were predicted as likely binding conformations by Haddock (Fig. 6A), with the chemical shift perturbations mapped onto the structure matching the predicted binding interface (Fig. 6B). Together the NMR mapping of ALYREF and KSHV ORF57 indicated that the viral protein contains two ALYREF binding sites, which apparently both interacted with the RRM domain. How these sites cooperate within the context of full-length proteins remains to be established.

### Mapping the interaction of UIF with KSHV ORF57

UIF and KSHV ORF57 are known binding partners^54^, however which regions contribute to these interactions has not been established. Here to investigate if the N-terminal ORF57 construct ksORF57^68–178^ interacts with UIF^NT^ we compared ORF57 HSQC spectra in the presence and absence of UIF^NT^. A large number of signals within this ksORF57^68–178^ construct were broadened, suggestive of an interaction which involves extended sites (Fig. 5Aii,B). The region overlapped with the shorter ALYREF binding site identified above (Fig. 5B), indicating that similar to ICP27, the binding sites for ALYREF and UIF within the intrinsically-disordered region of ksORF57 partially overlap.

## Discussion

Here, using *in vivo* and *in vitro* studies we dissected and mapped the interactions of homologous ICP27-like herpes virus proteins with cellular export factors ALYREF and UIF. We identified a novel interaction between the cellular mRNA export adaptor UIF and the multifunctional HSV-1 protein ICP27. Co-immunoprecipitation experiments in wild type HSV-1 KOS infected cells demonstrated that UIF interacted with WT-ICP27 (Fig. 2). Previously we identified by NMR an ALYREF binding motif in ICP27 consisting of residues 103–112, and later demonstrated that the site is important for efficient export of viral mRNA by mutating the main interacting residues W105, R107 and L108 to alanine (the ‘WRL’ mutant)^39,40^. The same ICP27 WRL mutant that is unable to bind ALYREF was still able to interact with UIF, which was suggestive of non-equivalent binding sites. The *in vivo* experiments also indicated that the N-terminal region of UIF (UIF^NT^) was sufficient for interaction with ICP27. Although the predictions based on primary sequence analysis indicated that the UIF^NT^ region should be largely unstructured and the C-terminal region UIF^CT^ expected to be globular, in contrast we observed by NMR evidence of a globular folded protein within full length and UIF^NT^ but not the isolated C-terminal UIF^CT^ region (Fig. 1). We speculate that the identified folded domain found within UIF^NT^ may be responsible for the interaction with ICP27. Despite these investigations of UIF folding, we cannot discount that the UIF fragments chosen may be perturbed by the truncation and the C-terminal domain of UIF may be folded *in vivo*. Indeed, it is likely that the UIF C-terminal domain contributes to the global protein fold, as its removal caused significant signal changes in the upfield region of the NMR spectra. In order to identify a possible UIF^NT^ interaction site within the intrinsically disordered N-terminus of ICP27, we used NMR signal perturbation mapping which identified the regions aa104–111 and 124–128 to be involved in UIF interaction (Fig. 3). The UIF binding motif also overlaps with the nuclear localization sequence of ICP27 but any functional consequence of this is unknown.

The ICP27 homolog ORF57 from KSHV (ksORF57) was the first viral protein identified as a UIF binding partner, and it also can interact with ALYREF and other cellular proteins involved in mRNA maturation^50,53–55^. We used solution NMR spectroscopy to investigate the ksORF57 interactions with both ALYREF and UIF. The established ALYREF binding site on ksORF57 was previously identified by deletion mutagenesis by Malik *et al*.^50^. These pioneering studies indicated the flexible N- and C-terminal domains of ALYREF interacted with residues 181–215 of ksORF57, the latter is now known to be part of the globular domain of ksORF57. Deletion analysis within folded domains runs a risk of perturbing their tertiary structure; therefore, careful experiments were performed here avoiding deletions within folded domains to preclude structure perturbations while mapping the interaction site. We obtained NMR data indicative of the presence of an interaction site between the RRM of ALYREF and ksORF57Δ153 (Fig. 4B), which includes the previously identified ALYREF site suggested via mutation of a PxxP motif (residues 208–211)^51^. Interestingly other ALYREF binding sites have been identified in N-terminal intrinsically disordered regions (IDRs) of HSV-1 ICP27 and HVS ORF57, which are distant and close homologs respectively of ksORF57^38,49,50^. We therefore investigated a possible second ALYREF binding site within the predicted N-terminal IDR of ksORF57 by NMR. The data indicated a short ALYREF binding site of ORF57 aa126–134 within a construct comprising ksORF57 residues 68–178 (Fig. 5). NMR experiments also indicated ksORF57^68–178^ construct interacted with the RRM domain of REF (Fig. 4). It was possible to construct a structural model of the ksORF57-ALYREF interaction, which revealed a ksORF57 α-helix binding to the α-helical face of the RRM domain (Fig. 6), similar to that previously determined with higher precision for HVS ORF57 binding to ALYREF^40,48^. Our NMR mapping results corroborate the earlier finding that N-terminal IDR of ksORF57 containing NLS also contributes to the binding with ALYREF^52^. As the UIF interaction site on ksORF57 was unknown, we therefore explored the possibility of UIF binding within the N-terminal IDR of ksORF57 using solution NMR signal perturbation mapping. The experiments indicated an extended UIF^NT^ binding site within the ksORF57^68–178^ construct (Fig. 5).

Together our results therefore indicated that ksORF57 contains two binding motifs able to interact with the RRM-domain of ALYREF. Previous studies may not have located the viral IDR interaction site with the ALYREF RRM due to destabilizing truncations of the cellular protein. Specifically a construct comprising REF2-I residues 74–152 did not bind ORF57^50^, however in subsequent studies a nearly identical construct comprising REF2-I residues 71–155 was shown by NMR to be structurally destabilized, and the minimal region containing the stable RRM domain was residues 54–155 (REFΔ53)^21^. Although implied previously from mutagenesis studies of N-terminal region of ksORF57^52^, involvement of N-terminal region of ksORF57 in interaction with ALYREF may be not as strong as the interaction mediated by C-terminal domain, explaining the deleterious effect of PxxP motif deletion on protein binding^51^. However, the two separate binding sites identified here by NMR should contribute synergistically to the overall molecular binding. Our NMR mapping experiments also indicated an interaction between the UIF^NT^ and ksORF57 N-terminal IDR. For this region the identified binding sites for UIF^NT^ and ALYREF partially overlap, a feature in common with the binding sites identified here on ICP27 (Figs 3,5).

The proteins ALYREF and UIF provide redundancy in mRNA nuclear export, a process that utilizes multiple protein-protein and protein-RNA interactions to mediate the processing and passage of the nascent transcript. A characteristic revealed by detailed studies of a number of these interactions, such as those of UAP56, RNA and TAP/NXF1 with ALYREF, is overlapping binding sites that mediate the complex passage of RNA from one protein to another. We have also observed a similar mechanism in HVS ORF57 for interaction with ALYREF and viral RNA^48^. The ORF57 interactions with UIF and ALYREF appeared competitive^54^. Here we provide experimental evidence of overlapping yet non-equivalent binding sites, which provides an explanation for this competition. Similarly, our NMR data indicated overlapping ALYREF and UIF binding motifs on ICP27 and we provide corroborative *in vivo* data for the ICP27 interaction with UIF, which was previously unidentified. Together the data suggest a degree of conservation of binding modes for the cellular proteins UIF and ALYREF within ICP27 and ORF57 despite a lack of obvious sequence similarity. As many interactions studied here occur predominately within the N-terminal IDRs of ORF57 and ICP27, and these proteins form stable homo-dimers, it is possible that they can bind UIF and ALYREF simultaneously one via each monomer. The ability of ICP27 and ORF57 to interact with different cellular mRNA export factors allows the virus to exploit the inherent redundancy within their hosts and improve the efficiency of viral mRNA export. Targeting these multiple mRNA export factor interactions as part of an antiviral therapy would be challenging; however the inhibition of UAP56 catalysis provides a pinch point to block TREX assembly and a more credible method for combating herpesvirus lytic infection^59^. The data and binding motifs identified in the current study will aid the exploration and improve our understanding of the complex mechanistic details of these multi-protein, multi-interaction assemblies that facilitate mRNA maturation.

## Materials and Methods

### Immunoprecipitation and western blot analysis

HeLa cells were grown in Dulbecco’s modified Eagle’s medium (DMEM, Thermo Scientific) supplemented with 10% newborn calf serum (Life Technologies). Cells were infected with wild type HSV-1 KOS or mutant virus WRL or ΔRGG^39^ as indicated in the figure legend at a multiplicity of infection of 10. Transfection of plasmid DNA was performed by using Lipofectamine 2000 reagent (Life Technologies) according to the manufacturer’s protocol. Cells were infected 24 h after transfection. Cells were lysed at 8 h after infection in low salt lysis buffer (10 mM Tris pH 7.4, 3 mM CaCl_2_, 2 mM MgCl_2_, 0.5% NP-40 and protease inhibitor cocktail (Roche)). The cell lysate was passed through a syringe with a 25-gauge needle ten times. The nuclei were pelleted by centrifugation at 14,000 g for 30 s. The supernatant was transferred to a new tube as the cytoplasmic fraction. The nuclear pellet was resuspended in high salt extraction buffer consisting of PBS containing 250 mM NaCl, 0.5% NP-40, and protease inhibitor cocktail and combined with the cytoplasmic fraction. Immunoprecipitations were performed with anti-ICP27 monoclonal antibody P1119 (Virusys) using Dynabeads protein-G magnetic beads (Life Technologies) according to the manufacturer’s protocol. Protein samples were fractionated on 10% SDS-polyacrylamide gels and transferred to nitrocellulose membrane. Blots were probed with anti-ICP27 antibody or anti-Flag antibody (Sigma) and analyzed by SuperSignal chemiluminescent substrate (Thermo Scientific).

### Protein expression and purification

UIF and ORF57 constructs were obtained by gene synthesis with codon optimization for expression in *E*. *coli* (Thermofisher). KSHV ORF57 residues 68–178 (ksORF57^68–178^) and three regions coding homo sapiens UIF (Uniprot ref: Q96QD9) residues 1-318 (full-length), 1–190 and 177–318, here named UIF^FL^, UIF^NT^ and UIF^CT^ respectively, were each ligated into the expression plasmid pET-15b via the NdeI and XhoI restriction sites resulting in constructs with an N-terminal His_6_-tag to facilitate purification. DNA coding for KSHV ORF57 residues 153–455 (here named ksORF57Δ153) was ligated into pET-21a in the BamHI and XhoI restriction sites with an N-terminal thioredoxin solubility tag, the two domains were connected with a strep-II-tag and HRV-3C protease cleavage site. UIF and ORF57 proteins were expressed in T7 express *E*. *coli* (NEB) in terrific broth or M9 minimal media supplemented with stable isotopes where appropriate. Cells were cultured at 37 °C until an OD_600_ of 0.6 was reached and then the temperature was decreased to 20 °C prior to induction with 0.25 mM IPTG, incubation was then continued for 16 h and then cells were harvested by centrifugation (5,000 g, 20 m). The ksORF57Δ153 protein was purified with affinity strep-tag resin then cleaved by HRV-3C protease and purified by size exclusion chromatography as previously described for ICP27Δ241^25^. The UIF^FL^, UIF^NT^ and ^15^N labeled ksORF57^68–178^ constructs were purified by the same method – pellets were resuspended in running buffer (RB: 50 mM Tris, 0.5 M NaCl, 50 mM L-Arg, 50 mM L-Glu, pH 8.0) plus 0.5% Triton X-100, DNase and protease inhibitor cocktail, then lysed on wet ice by sonication. Lysate was centrifuged (35,000 g, 30 m, 4 °C) and supernatant filtered through 0.2 µm prior to binding to TALON purification resin (Clontech) in a gravity flow column equilibrated in RB. Resin was washed with RB plus 5 mM imidazole and protein was eluted with 200 mM imidazole in RB. The sample was purified further by size exclusion chromatography using a Superdex 75 26/600 column equilibrated in GF buffer: 20 mM sodium phosphate, 150 mM NaCl, 50 mM L-Arg, 50 mM L-Glu, 1 mM EDTA, 1 mM TCEP, pH 6.2. For unlabeled ksORF57^68–178^ the purification was carried out using TALON purification as described above with an additional wash with 5 mM ATP added to the buffer prior to elution to remove residual chaperone contamination. Then further purified by ion exchange; protein was dialyzed into low salt buffer (20 mM HEPES, 40 mM NaCl, 1 mM TCEP, pH 7.4) and loaded onto a HiTrap Q Sepharose column washed with low salt buffer then eluted using a linear gradient into high salt buffer (20 mM HEPES, 1 M NaCl, 1 mM TCEP, pH 7.4). UIF^CT^ was expressed in the insoluble fraction, therefore cells were lysed and centrifuged as above, then the pellet was resuspended in denaturing buffer (DB: 50 mM Tris, 6 M Guanidine hydrochloride, 1 M NaCl, pH 8.0) over 16 h. The solution was centrifuged (35,000 g, 30 m, 16 °C) and the supernatant bound onto TALON resin equilibrated in DB. Resin was washed with 5 mM imidazole in DB, and protein eluted in 200 mM imidazole in DB. UIF^CT^ was then refolded/solubilized in non-denaturing conditions by rapid 25-fold dilution into ice cold refolding buffer (50 mM Tris, 0.5 M L-Arg, 5 mM DTT, 1 mM EDTA, pH 8.0), then dialyzed in 3.5 kDa snakeskin dialysis membrane vs 20 mM Tris, 2 mM DTT, 1 mM EDTA, pH 8.0. The expression and purification of ICP27 constructs coding residues 103–155, 1–138 and ICP27Δ241, and of the murine variant of ALYREF, REF2-I comprising 54–155 (REFΔ53), 1-155 (REF^1–155^) and 156–218 (REF-C), have been described previously^21,25,40^.

### NMR spectroscopy

Prior to NMR studies proteins were dialyzed into NMR buffer (20 mM sodium phosphate, 50 mM NaCl, 50 mM L-Arg, 50 mM L-Glu, 1 mM EDTA, 2 mM TCEP, pH 6.2) unless stated otherwise, and concentrated using Vivaspin centrifugal devices. L-Arg/L-Glu was added to improve protein sample solubility and stability^60^. EDTA concentration was decreased to 0.1 mM for titrations involving constructs ICP27Δ241 and ORF57Δ153 to avoid removal of bound Zn^2+^ ions needed for structural integrity of IHDs. 5% v/v D_2_O was added to samples for lock and data was acquired at 298 K on a Bruker Avance III 800 MHz and Avance 600 MHz spectrometers equipped with cryoprobes. Topspin 3.5 was used for data acquisition and processing. Three backbone amide signal assignments were carried out: firstly, for ksORF57^68–178^ uniformly ^15^N labeled protein concentrated to 0.4 mM was used for the acquisition of 3D TOCSY-HSQC and NOESY-HSQC spectra with mixing times of 60 ms and 120 ms respectively. Secondly for ICP27^1–138^ a uniformly ^13^C,^15^N labeled protein sample concentrated to 1.0 mM was used for the acquisition of triple resonance 3D spectra HNCO, HN(CA)CO, HNCA CBCA(CO)NH and HNCACB. Thirdly, uniformly ^13^C,^15^N labeled ICP27^103–155^ concentrated to 0.45 mM (buffer: 20 mM phosphate, 50 mM NaCl, 50 mM L-Arg/L-Glu/β-mercaptoethanol and 10 mM EDTA, pH 6.2) was used for the acquisition of 3D triple resonance spectra HNCO, HN(CA)CO, HNCA CBCA(CO)NH and HNCACB. In the latter, backbone amide signals positions for residues 103–137 were identical to those previously determined for ICP27^103–138^, the triple resonance data confirmed the previous results^40^ and allowed assignment of the RGG-box. Mapping of binding sites was carried out by acquisition of HSQC spectra of uniformly ^15^N labeled protein without binding partner and addition of a small volume of unlabeled potential binding partner from a concentrated stock in matching buffer resulting in an equimolar mixture of the two species. A second HSQC spectrum of the bound state was then acquired for comparison with the free spectra. Protein concentrations were ca. 50 µM, details for each experiment are shown in Supplementary Information Table S1. The distance of signal movements were measured as described previously for the ICP27-REF^1–155^ interaction^40^, signal shifts greater than 1 standard deviation of all peak movement distances (1σ) were judged ‘moderate’ and greater than 2σ ‘large’. Peak heights were also measured and compared between bound and free states. A loss in intensity due to broadening was labeled significant if the signal loss was greater than 75%. All spectral assignment and analysis was carried out using Sparky^61^.

### Structural model of the REFΔ53-ksORF57^68–178^ interaction

The Haddock web server was used to generate a structural model for the interaction between ksORF57^68–178^ and REFΔ53^58^. As starting structures, the coordinates of REFΔ53 from the PDB 2YKA were used, and an *ab initio* model of ksORF57 model of residues 123–140 was generated by PHYRE2^62^. Using the Haddock ‘expert’ interface active residues were defined as 84, 86, 87, 91–98, 102, 120, 134, 135, 137–143, 146 plus 147 for REF and 126–134 for ORF57, passive residues were defined automatically for REF and residues 125, 135, 136, 137 for ORF57. In addition, the REF RRM domain loops (residues 81–86, 109–113, 138–145) were defined as semi-flexible, and the N- and C- termini (40–74, 152–163) fully flexible. In ORF57 central α-helical residues 128–137 were semi-flexible and 123–127, 138–140 were fully flexible. The lowest energy models generated was analyzed in Chimera^63^.

## Electronic supplementary material

Supplementary Information

## References

[1] Roeder RG (1996). The role of general initiation factors in transcription by RNA polymerase II. Trends Biochem Sci.

[2] Sonenberg N, Hinnebusch AG (2009). Regulation of translation initiation in eukaryotes: mechanisms and biological targets. Cell.

[3] Cheng H (2006). Human mRNA export machinery recruited to the 5′ end of mRNA. Cell.

[4] Girard C (2012). Post-transcriptional spliceosomes are retained in nuclear speckles until splicing completion. Nat Commun.

[5] Proudfoot NJ (2011). Ending the message: poly(A) signals then and now. Genes Dev.

[6] Wickramasinghe VO, Laskey RA (2015). Control of mammalian gene expression by selective mRNA export. Nat Rev Mol Cell Biol.

[7] Masuda S (2005). Recruitment of the human TREX complex to mRNA during splicing. Genes Dev.

[8] Heath CG, Viphakone N, Wilson SA (2016). The role of TREX in gene expression and disease. Biochem J.

[9] Zhou Z (2000). The protein Aly links pre-messenger-RNA splicing to nuclear export in metazoans. Nature.

[10] Luo ML (2001). Pre-mRNA splicing and mRNA export linked by direct interactions between UAP56 and Aly. Nature.

[11] Rodrigues JP (2001). REF proteins mediate the export of spliced and unspliced mRNAs from the nucleus. Proc Natl Acad Sci USA.

[12] Shen H (2008). Distinct activities of the DExD/H-box splicing factor hUAP56 facilitate stepwise assembly of the spliceosome. Genes Dev.

[13] Shen J, Zhang L, Zhao R (2007). Biochemical characterization of the ATPase and helicase activity of UAP56, an essential pre-mRNA splicing and mRNA export factor. J Biol Chem.

[14] Strasser K (2002). TREX is a conserved complex coupling transcription with messenger RNA export. Nature.

[15] Taniguchi I, Ohno M (2008). ATP-dependent recruitment of export factor Aly/REF onto intronless mRNAs by RNA helicase UAP56. Mol Cell Biol.

[16] Hautbergue GM, Hung ML, Golovanov AP, Lian LY, Wilson SA (2008). Mutually exclusive interactions drive handover of mRNA from export adaptors to TAP. Proc Natl Acad Sci USA.

[17] Hautbergue GM (2009). UIF, a New mRNA export adaptor that works together with REF/ALY, requires FACT for recruitment to mRNA. Curr Biol.

[18] Chang CT (2013). Chtop is a component of the dynamic TREX mRNA export complex. EMBO J.

[19] Dufu K (2010). ATP is required for interactions between UAP56 and two conserved mRNA export proteins, Aly and CIP29, to assemble the TREX complex. Genes Dev.

[20] Folco EG, Lee CS, Dufu K, Yamazaki T, Reed R (2012). The proteins PDIP3 and ZC11A associate with the human TREX complex in an ATP-dependent manner and function in mRNA export. PLoS One.

[21] Golovanov AP, Hautbergue GM, Tintaru AM, Lian LY, Wilson SA (2006). The solution structure of REF2-I reveals interdomain interactions and regions involved in binding mRNA export factors and RNA. RNA.

[22] Perez-Alvarado GC (2003). Structure of the nuclear factor ALY: insights into post-transcriptional regulatory and mRNA nuclear export processes. Biochemistry.

[23] Sandri-Goldin RM (2011). The many roles of the highly interactive HSV protein ICP27, a key regulator of infection. Future Microbiol.

[24] Zhi Y, Sciabica KS, Sandri-Goldin RM (1999). Self-interaction of the herpes simplex virus type 1 regulatory protein ICP27. Virology.

[25] Tunnicliffe RB (2015). The structure of the folded domain from the signature multifunctional protein ICP27 from herpes simplex virus-1 reveals an intertwined dimer. Sci Rep.

[26] Patel V (2015). Structure of the C-Terminal Domain of the Multifunctional ICP27 Protein from Herpes Simplex Virus 1. J Virol.

[27] Majerciak V (2015). Stability of structured Kaposi’s sarcoma-associated herpesvirus ORF57 protein is regulated by protein phosphorylation and homodimerization. J Virol.

[28] Lischka P, Thomas M, Toth Z, Mueller R, Stamminger T (2007). Multimerization of human cytomegalovirus regulatory protein UL69 via a domain that is conserved within its herpesvirus homologues. J Gen Virol.

[29] Baudoux L, Defechereux P, Rentier B, Piette J (2000). Gene activation by Varicella-zoster virus IE4 protein requires its dimerization and involves both the arginine-rich sequence, the central part, and the carboxyl-terminal cysteine-rich region. J Biol Chem.

[30] Key SC, Yoshizaki T, Pagano JS (1998). The Epstein-Barr virus (EBV) SM protein enhances pre-mRNA processing of the EBV DNA polymerase transcript. J Virol.

[31] Tunnicliffe, R. B., Collins, R. F., Ruiz Nivia, H. D., Sandri-Goldin, R. M. & Golovanov, A. P. The ICP27 Homology Domain of the Human Cytomegalovirus Protein UL69 Adopts a Dimer-of-Dimers Structure. *M Bio* **9** (2018). 10.1128/mBio.01112-18 PMC6016253 29921674

[32] Corbin-Lickfett KA, Chen IH, Cocco MJ, Sandri-Goldin RM (2009). The HSV-1 ICP27 RGG box specifically binds flexible, GC-rich sequences but not G-quartet structures. Nucleic Acids Res.

[33] Mears WE, Rice SA (1996). The RGG box motif of the herpes simplex virus ICP27 protein mediates an RNA-binding activity and determines *in vivo* methylation. J Virol.

[34] Sandri-Goldin RM (1998). ICP27 mediates HSV RNA export by shuttling through a leucine-rich nuclear export signal and binding viral intronless RNAs through an RGG motif. Genes Dev.

[35] Mears WE, Lam V, Rice SA (1995). Identification of nuclear and nucleolar localization signals in the herpes simplex virus regulatory protein ICP27. J Virol.

[36] Soliman TM, Sandri-Goldin RM, Silverstein SJ (1997). Shuttling of the herpes simplex virus type 1 regulatory protein ICP27 between the nucleus and cytoplasm mediates the expression of late proteins. J Virol.

[37] Chen IH, Li L, Silva L, Sandri-Goldin RM (2005). ICP27 recruits Aly/REF but not TAP/NXF1 to herpes simplex virus type 1 transcription sites although TAP/NXF1 is required for ICP27 export. J Virol.

[38] Chen IH, Sciabica KS, Sandri-Goldin RM (2002). ICP27 interacts with the RNA export factor Aly/REF to direct herpes simplex virus type 1 intronless mRNAs to the TAP export pathway. J Virol.

[39] Tian X, Devi-Rao G, Golovanov AP, Sandri-Goldin RM (2013). The interaction of the cellular export adaptor protein Aly/REF with ICP27 contributes to the efficiency of herpes simplex virus 1 mRNA export. J Virol.

[40] Tunnicliffe RB (2011). Structural basis for the recognition of cellular mRNA export factor REF by herpes viral proteins HSV-1 ICP27 and HVS ORF57. PLoS Pathog.

[41] Dai-Ju JQ, Li L, Johnson LA, Sandri-Goldin RM (2006). ICP27 interacts with the C-terminal domain of RNA polymerase II and facilitates its recruitment to herpes simplex virus 1 transcription sites, where it undergoes proteasomal degradation during infection. J Virol.

[42] Hernandez, F. P. & Sandri-Goldin, R. M. Head-to-tail intramolecular interaction of herpes simplex virus type 1 regulatory protein ICP27 is important for its interaction with cellular mRNA export receptor TAP/NXF1. *MBio* **1** (2010). 10.1128/mBio.00268-10 PMC2975367 21060739

[43] Sciabica KS, Dai QJ, Sandri-Goldin RM (2003). ICP27 interacts with SRPK1 to mediate HSV splicing inhibition by altering SR protein phosphorylation. EMBO J.

[44] Bryant HE, Wadd SE, Lamond AI, Silverstein SJ, Clements JB (2001). Herpes simplex virus IE63 (ICP27) protein interacts with spliceosome-associated protein 145 and inhibits splicing prior to the first catalytic step. J Virol.

[45] Schumann S, Jackson BR, Baquero-Perez B, Whitehouse A (2013). Kaposi’s sarcoma-associated herpesvirus ORF57 protein: exploiting all stages of viral mRNA processing. Viruses.

[46] Majerciak V, Zheng ZM (2015). KSHV ORF57, a protein of many faces. Viruses.

[47] Boyne JR, Colgan KJ, Whitehouse A (2008). Herpesvirus saimiri ORF57: a post-transcriptional regulatory protein. Front Biosci.

[48] Tunnicliffe RB, Hautbergue GM, Wilson SA, Kalra P, Golovanov AP (2014). Competitive and cooperative interactions mediate RNA transfer from herpesvirus saimiri ORF57 to the mammalian export adaptor ALYREF. PLoS Pathog.

[49] Williams BJ (2005). The prototype gamma-2 herpesvirus nucleocytoplasmic shuttling protein, ORF 57, transports viral RNA through the cellular mRNA export pathway. Biochem J.

[50] Malik P, Blackbourn DJ, Clements JB (2004). The evolutionarily conserved Kaposi’s sarcoma-associated herpesvirus ORF57 protein interacts with REF protein and acts as an RNA export factor. J Biol Chem.

[51] Boyne JR, Colgan KJ, Whitehouse A (2008). Recruitment of the complete hTREX complex is required for Kaposi’s sarcoma-associated herpesvirus intronless mRNA nuclear export and virus replication. PLoS Pathog.

[52] Majerciak V, Yamanegi K, Nie SH, Zheng ZM (2006). Structural and functional analyses of Kaposi sarcoma-associated herpesvirus ORF57 nuclear localization signals in living cells. J Biol Chem.

[53] Majerciak V (2011). Kaposi’s sarcoma-associated herpesvirus ORF57 interacts with cellular RNA export cofactors RBM15 and OTT3 to promote expression of viral ORF59. J Virol.

[54] Jackson BR (2011). An interaction between KSHV ORF57 and UIF provides mRNA-adaptor redundancy in herpesvirus intronless mRNA export. PLoS Pathog.

[55] Schumann S, Baquero-Perez B, Whitehouse A (2016). Interactions between KSHV ORF57 and the novel human TREX proteins, CHTOP and CIP29. J Gen Virol.

[56] Yachdav G (2014). PredictProtein–an open resource for online prediction of protein structural and functional features. Nucleic Acids Res.

[57] Stutz F (2000). REF, an evolutionary conserved family of hnRNP-like proteins, interacts with TAP/Mex67p and participates in mRNA nuclear export. RNA.

[58] van Zundert GCP (2016). The HADDOCK2.2 Web Server: User-Friendly Integrative Modeling of Biomolecular Complexes. J Mol Biol.

[59] Schumann S (2016). Targeting the ATP-dependent formation of herpesvirus ribonucleoprotein particle assembly as an antiviral approach. Nat Microbiol.

[60] Golovanov AP, Hautbergue GM, Wilson SA, Lian LY (2004). A simple method for improving protein solubility and long-term stability. J Am Chem Soc.

[61] Lee W, Tonelli M, Markley JL (2015). NMRFAM-SPARKY: enhanced software for biomolecular NMR spectroscopy. Bioinformatics.

[62] Kelley LA, Mezulis S, Yates CM, Wass MN, Sternberg MJ (2015). The Phyre2 web portal for protein modeling, prediction and analysis. Nat Protoc.

[63] Pettersen EF (2004). UCSF Chimera–a visualization system for exploratory research and analysis. J Comput Chem.

