# Supplementary material for: Overlapping motifs on the herpes viral proteins ICP27 and ORF57 mediate interactions with the mRNA export adaptors ALYREF and UIF
Source: Sci Rep. 2018 Oct 9;8:15005. doi: 10.1038/s41598-018-33379-x (PMC6177440; doi:10.1038/s41598-018-33379-x)
Supplement: Supplementary file 1 — Supplementary Information [file 41598_2018_33379_MOESM1_ESM.pdf]

Supplementary Information

Overlapping motifs on the herpes viral proteins ICP27 and ORF57 mediate interactions with the mRNA export adaptors ALYREF and UIF

Authors

Richard B. Tunnicliffe[1], Xiaochen Tian[2], Joanna Storer[1], Rozanne M. Sandri-Goldin[2], Alexander P. Golovanov[1]

Affiliations

[1] Manchester Institute of Biotechnology, School of Chemistry, Faculty of Science and Engineering, The University of Manchester, Manchester M1 7DN, UK.

[2] Department of Microbiology and Molecular Genetics, School of Medicine, University of California, Irvine, CA 92697-4025, USA.

Correspondence and requests for materials should be addressed to A.P.G (email: a.golovanov@manchester.ac.uk) or R.M.S.-G. (email rmsandri@uci.edu).

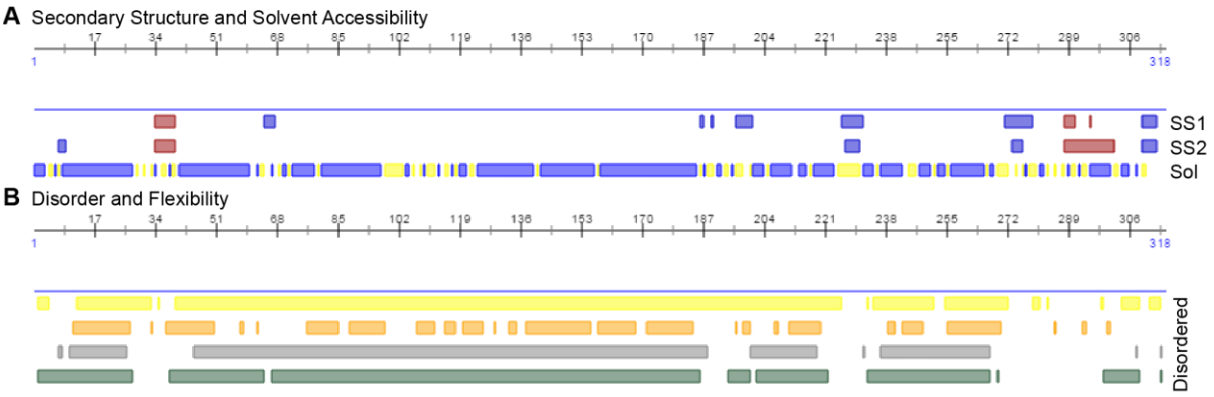

Figure S1. Primary sequence based predictions of UIF domains using the PredictProtein server[1]. (A) Secondary structure (SS) and solvent accessibility (Sol) as labeled. SS: α-helix are red blocks, β-sheet are blue blocks. Sol: Solvent accessible are blue blocks, buried residues are yellow blocks. SS1 produced using REFPROFsec, SS2 with PROFsec and Sol with PROFAcc algorithms[2]. (B) Regions of predicted disorder shown as filled blocks calculated using the algorithms PROFbval, Ucon, NORSnet, Meta-disorder colored yellow, orange, grey and green respectively[3].

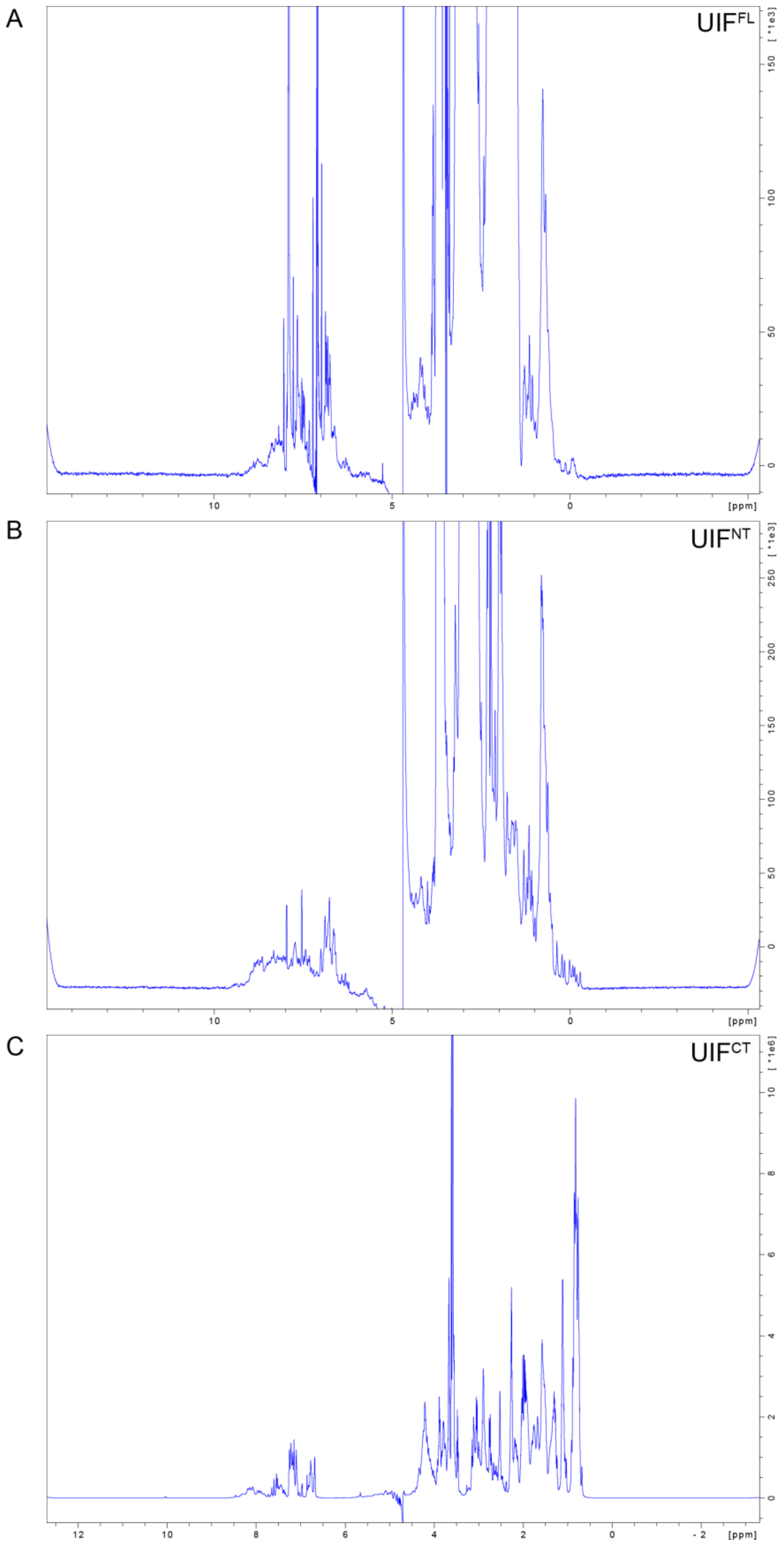

Figure S2. Full ^{1}H NMR spectra of UIF constructs shown in part on Fig. 2A. Sample conditions were (A) 90 µM UIF^{FL} in 20 mM HEPES, 150 mM NaCl, 50 mM Arg/Glu, 1 mM EDTA, 1 mM TCEP, pH 7.9. (B) 100 µM UIF^{NT} in 50 mM HEPES, 1 mM EDTA, 1 mM TCEP, pH 7.9. (C) 1.0 mM UIF^{CT} in 20 mM Tris, 2mM DTT, pH 8.0.

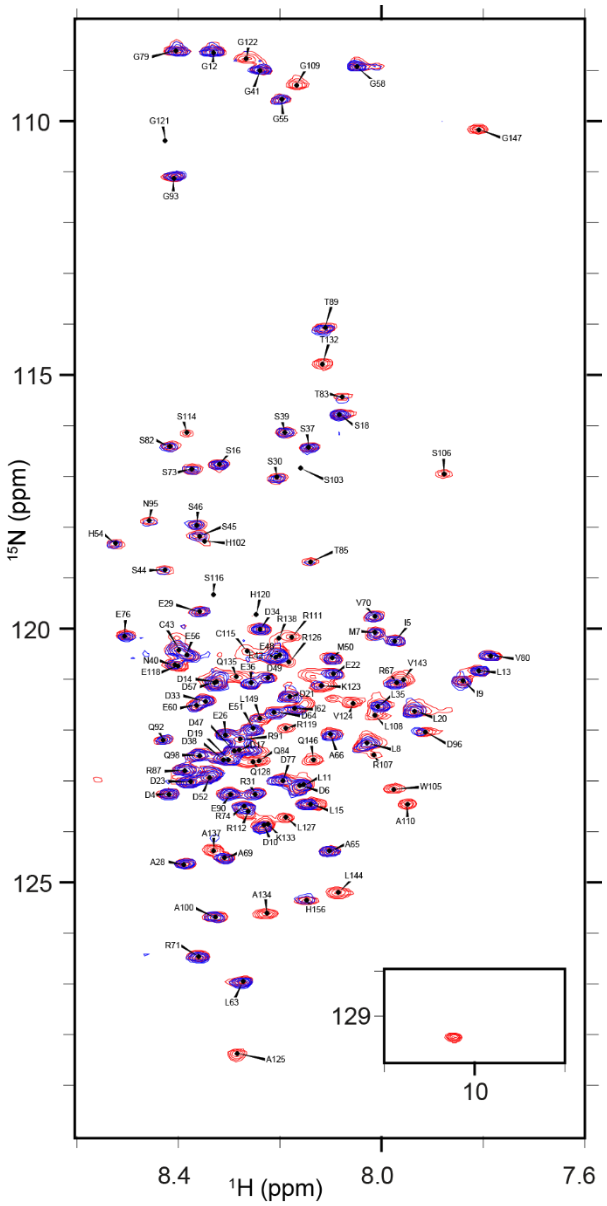

Figure S3. NMR mapping of UIF^{NT} interaction with ^{15}N-labelled ICP27$^{1\text{-}138}$. Free ICP27$^{1\text{-}138}$ is colored red and ICP27$^{1\text{-}138}$ with equimolar UIF^{NT} present is colored blue. Backbone amide signal assignments are labeled.

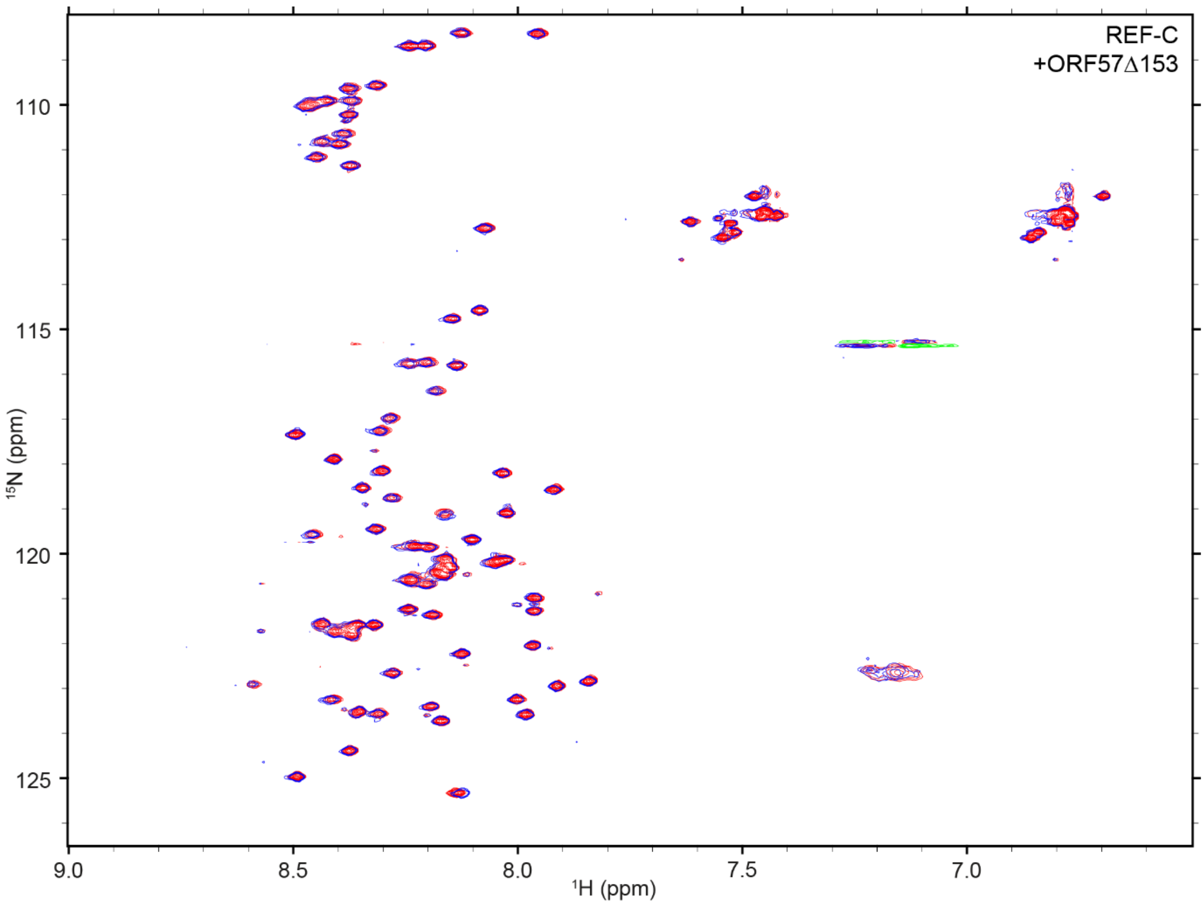

Figure S4. REF-C control spectra which indicated REF-C does not interact with ksORF57Δ153. HSQC spectra of ^{15}N labeled REF-C in presence and absence of unlabeled ksORF57Δ153 are colored blue and red respectively.

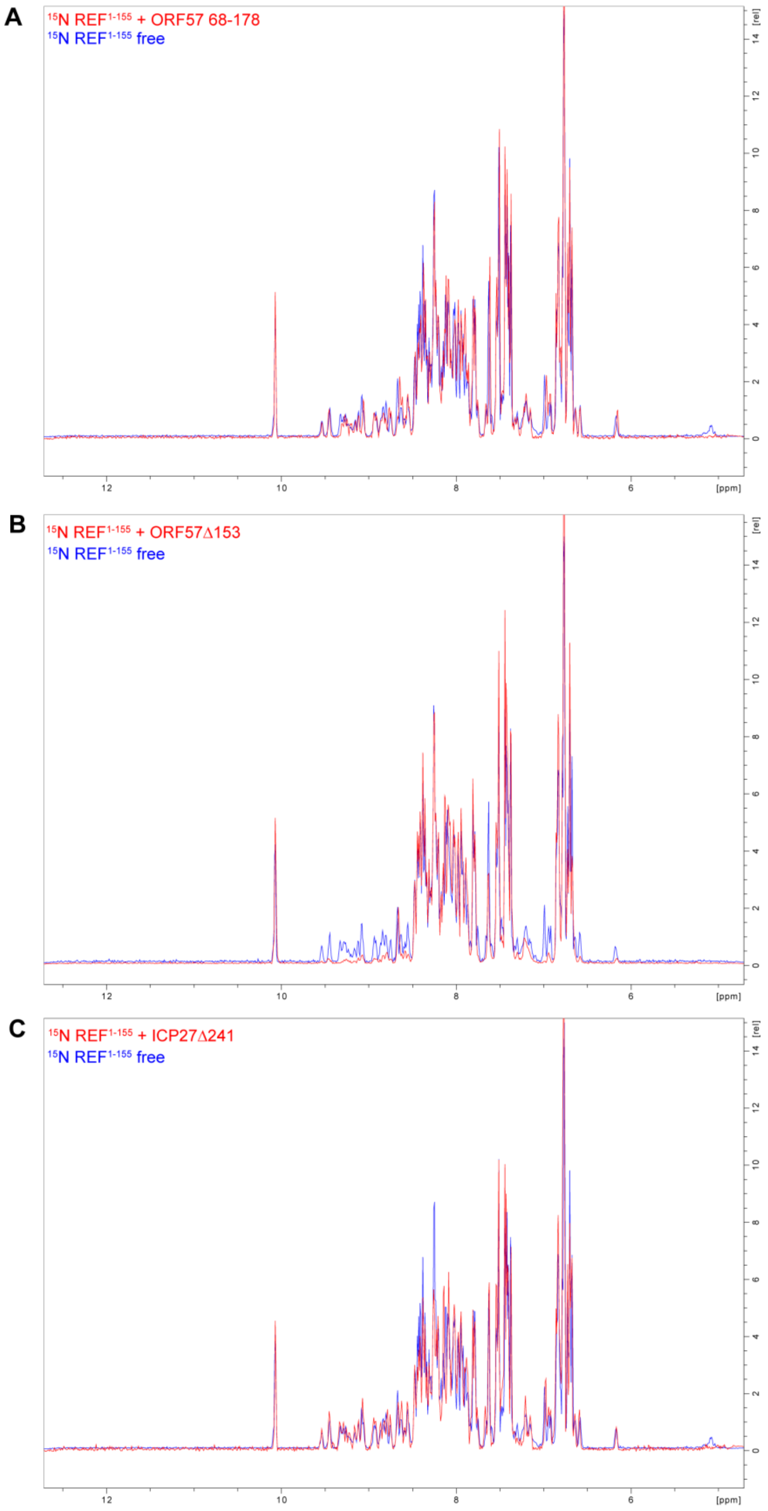

Figure S5. 1D ^{1}H projections of ^{15}N-HSQC spectra of REF$^{1-155}$. Free REF$^{1-155}$ in absence and presence of a potential binding partner as indicated are colored blue and red respectively: (A) ksORF57$^{68-178}$, (B) ksORF57Δ153 and (C) ICP27Δ241. Signal broadening in dispersed signals from the RRM domain of REF was observed in panel B indicative of an interaction with ksORF57Δ153.

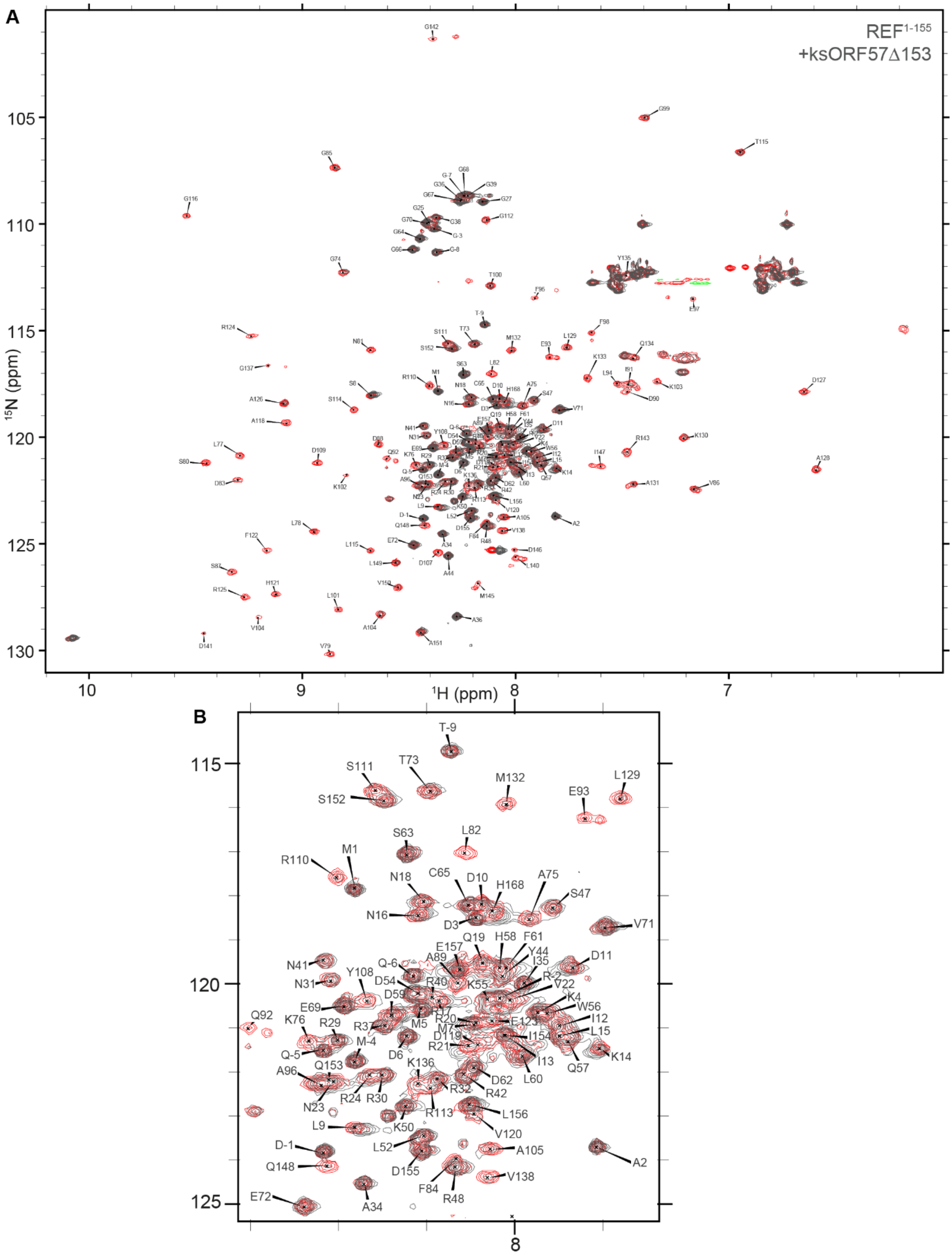

Figure S6. REF$^{1-155}$ interacts with ksORF57Δ153. Spectrum of free ^{15}N labeled REF$^{1-155}$ (grey) is overlaid with spectrum upon addition of equimolar unlabeled ksORF57Δ153 (red). The interaction induces significant peak broadening for residues in the folded RRM domain of REF, as well as minor signal shifts for its N-helix region caused by opening of REF conformation [4]. (A) Full spectra, (B) zoom of central region. The green signal is negative contours in free REF$^{1-155}$ spectra from Arginine in the buffer.

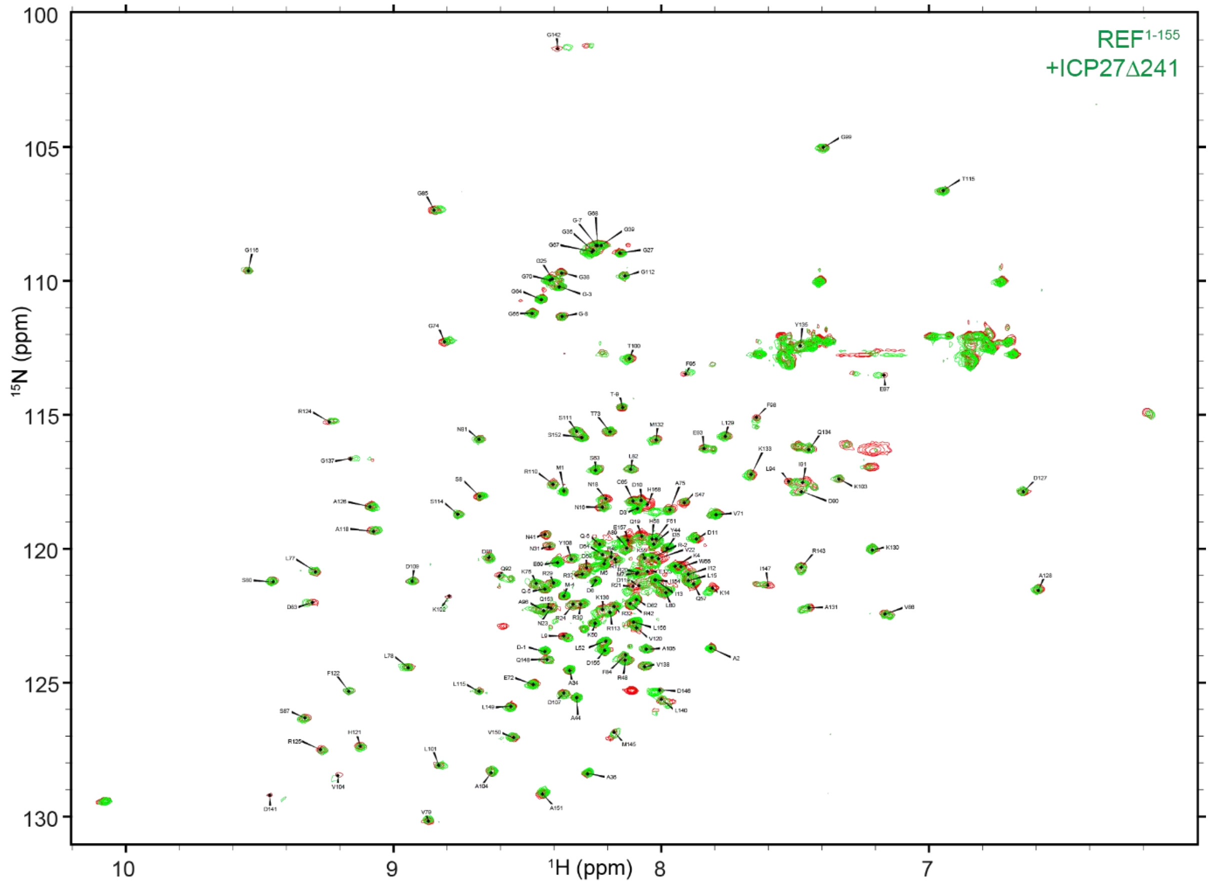

Figure S7. REF$^{1-155}$ does not interact with ICP27Δ241. Addition of equimolar unlabeled ICP27Δ241 to ^{15}N labeled REF$^{1-155}$ did not induce significant signal perturbations. Spectra colored red for REF$^{1-155}$ in isolation, and green for in the presence of ICP27Δ241.

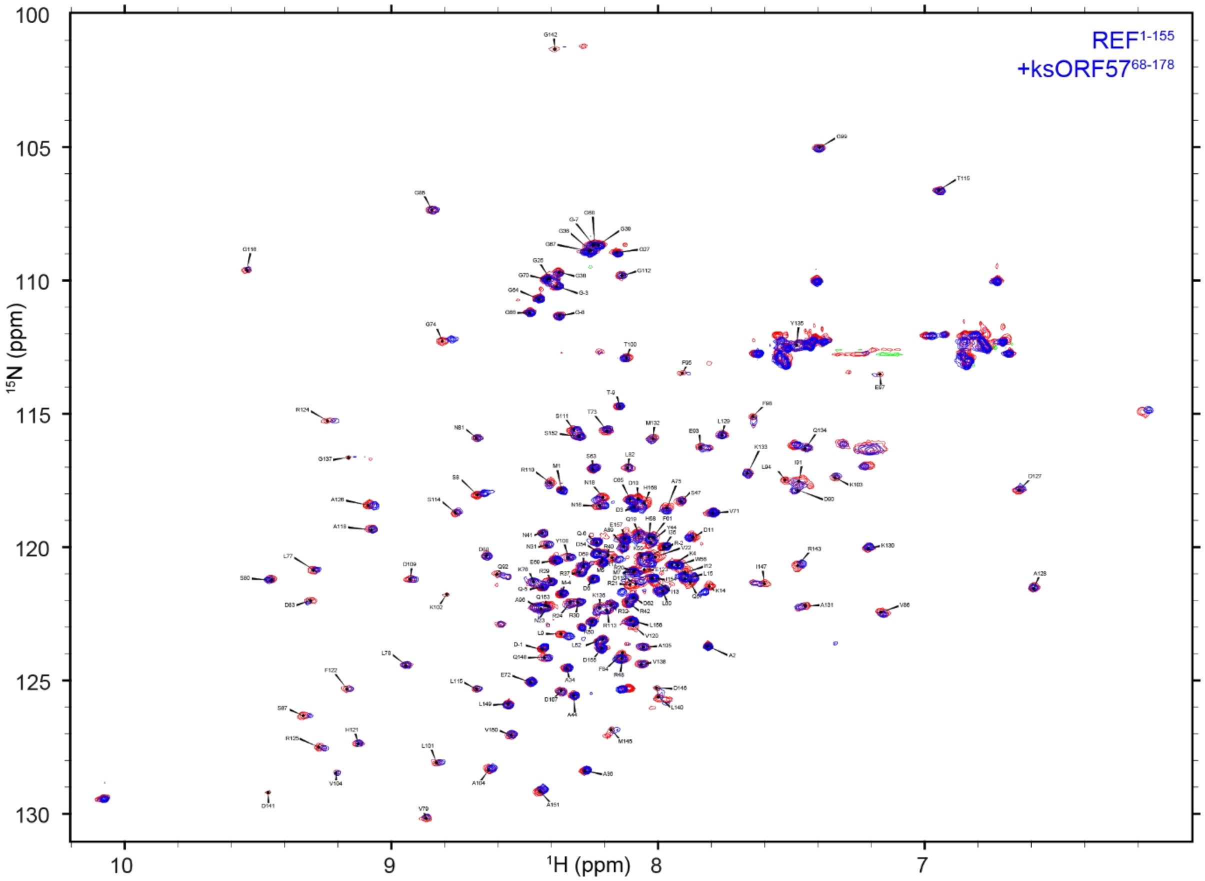

Figure S8. REF$^{1-155}$ interacts with ksORF57$^{68-178}$. Addition of equimolar unlabeled ksORF57$^{68-178}$ to ^{15}N labeled REF$^{1-155}$ resulted in signal perturbations within the dispersed RRM signals. Spectra colored red for REF$^{1-155}$ in isolation, and blue for in the presence of ksORF57$^{68-178}$.

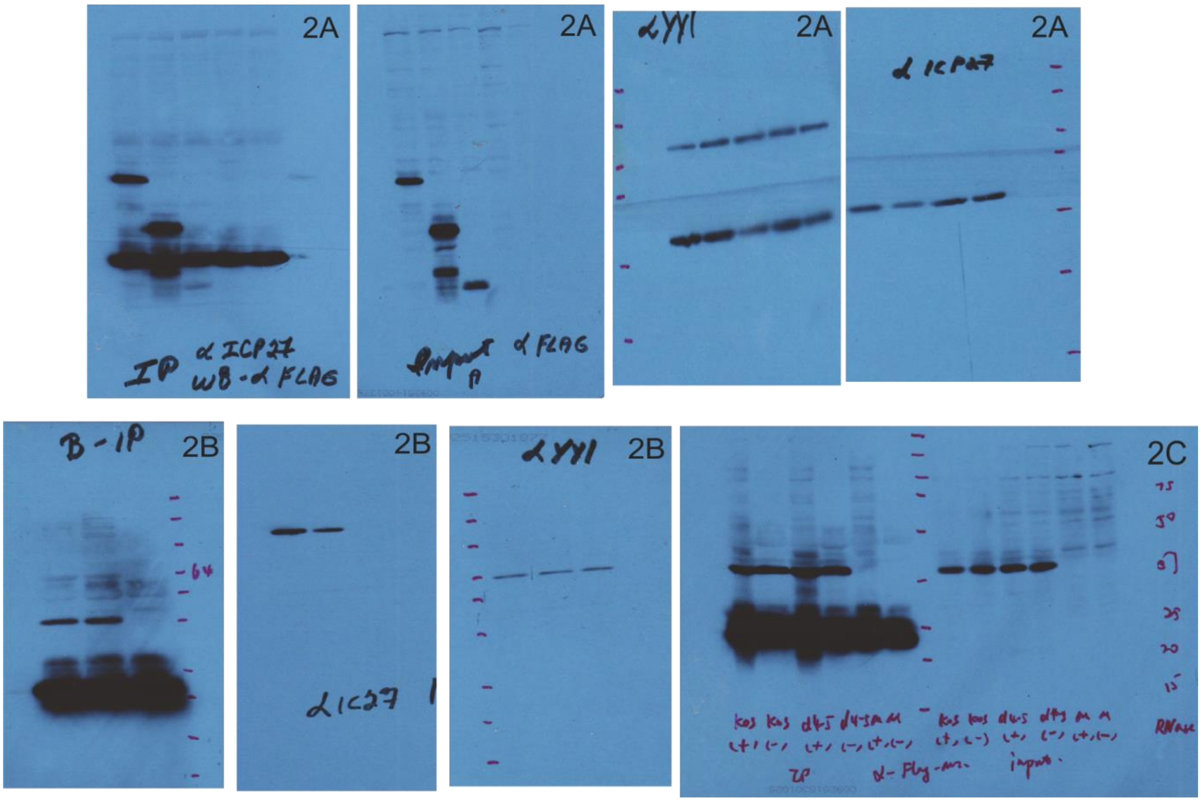

Figure S9. Original uncropped images of Western blots shown in Fig.2.

Table S1. Sample details for NMR interaction experiments. Concentrations indicated for equimolar combinations of proteins, one exception marked * was REFΔ53 + ksORF57 68-178 which were 125 µM + 625 µM respectively.

^{15}N labeled	Unlabeled binder	Concentration, µM	Spectrometer (MHz)
ICP27$^{1\text{-}138}$	UIF^{NT}	40	600
ICP27$^{103\text{-}155}$	UIF^{NT}	70	800
	REF$^{1\text{-}155}$	40	800
	REF$^{1\text{-}155}$ & UIF^{NT}	40	800
ksORF57$^{68\text{-}178}$	UIF^{NT}	50	800
	REF$^{1\text{-}155}$	50	800
	REF$^{1\text{-}155}$ & UIF^{NT}	50	800
*REFΔ53	ksORF57 $^{68\text{-}178}$	125 + 625	600
REF$^{1\text{-}155}$	ksORF57 $^{68\text{-}178}$	200	800
	ksORF57Δ153	88	800
	ICP27Δ241	90	800
REF-C	ksORF57Δ153	50	800

Supplemental References

1. Yachdav, G. et al. PredictProtein--an open resource for online prediction of protein structural and functional features. *Nucleic Acids Res* **42**, W337-43 (2014).
2. Rost, B. & Sander, C. Improved prediction of protein secondary structure by use of sequence profiles and neural networks. *Proc Natl Acad Sci U S A* **90**, 7558-62 (1993).
3. Schlessinger, A., Punta, M., Yachdav, G., Kajan, L. & Rost, B. Improved disorder prediction by combination of orthogonal approaches. *PLoS One* **4**, e4433 (2009).
4. Golovanov, A.P., Hautbergue, G.M., Tintaru, A.M., Lian, L.Y. & Wilson, S.A. The solution structure of REF2-I reveals interdomain interactions and regions involved in binding mRNA export factors and RNA. *RNA* **12**, 1933-48 (2006).
